# Conflict in the Intracellular Lives of Endosymbionts and Viruses: A Mechanistic Look at *Wolbachia*-Mediated Pathogen-blocking

**DOI:** 10.3390/v10040141

**Published:** 2018-03-21

**Authors:** Amelia R. I. Lindsey, Tamanash Bhattacharya, Irene L. G. Newton, Richard W. Hardy

**Affiliations:** Department of Biology, Indiana University, Bloomington, IN 47405, USA; amlind@indiana.edu (A.R.I.L.); tamanash@umail.iu.edu (T.B.)

**Keywords:** vector control, antiviral, symbiosis, endosymbiont, arbovirus, *Drosophila*, *Aedes*

## Abstract

At the forefront of vector control efforts are strategies that leverage host-microbe associations to reduce vectorial capacity. The most promising of these efforts employs *Wolbachia*, a maternally transmitted endosymbiotic bacterium naturally found in 40% of insects. *Wolbachia* can spread through a population of insects while simultaneously inhibiting the replication of viruses within its host. Despite successes in using *Wolbachia*-transfected mosquitoes to limit dengue, Zika, and chikungunya transmission, the mechanisms behind pathogen-blocking have not been fully characterized. Firstly, we discuss how *Wolbachia* and viruses both require specific host-derived structures, compounds, and processes to initiate and maintain infection. There is significant overlap in these requirements, and infection with either microbe often manifests as cellular stress, which may be a key component of *Wolbachia*’s anti-viral effect. Secondly, we discuss the current understanding of pathogen-blocking through this lens of cellular stress and develop a comprehensive view of how the lives of *Wolbachia* and viruses are fundamentally in conflict with each other. A thorough understanding of the genetic and cellular determinants of pathogen-blocking will significantly enhance the ability of vector control programs to deploy and maintain effective *Wolbachia*-mediated control measures.

## 1. Introduction

Viruses and arthropods are two of the most abundant and diverse branches of life. Some arthropods also function as important vectors for a wide assortment of RNA viruses that include the single-stranded positive sense virus families *Togaviridae* (e.g., chikungunya, Sindbis, Semliki Forest Viruses) and *Flaviviridae* (e.g., dengue, Zika, Japanese Encephalitis and West Nile Viruses)*,* negative sense *Bunyaviridae* (e.g., Rift Valley Fever Virus) and the double-stranded, segmented virus family *Reoviridae* (e.g., Blue Tongue Virus, Epizootic Hemorrhagic Fever Virus), representing an overwhelming number of virus-arthropod associations. These arthropod-borne viruses (arboviruses) represent significant global health concerns for humans and livestock. Additionally, increased frequency of global trade and travel has led to the expansion of arbovirus distributions. This increase in the incidence of viral epidemics in novel geographical locations is aided in part by virus adaptation to new vector and host species [1,2,3,4]. In certain cases, virus adaptation to these new environments correlates with increased disease severity and new clinical symptoms that pose significant socio-economic burdens on developing nations [5]. With the absence of conventional vaccines or antiviral drugs against arboviral diseases, and the high cost of personal repellents in developing countries, vector control remains the most effective tool for combating the spread of disease [6,7].

The symbiotic bacterium *Wolbachia* is an intracellular resident of a majority of insect species, currently undergoing testing as a vector control agent [8,9]. *Wolbachia* is transovarially transmitted from mother to offspring, and changes the physiology of its insect host to ensure faithful transmission each generation [10]. The ways in which *Wolbachia* alters host physiology have brought it into the forefront of vector control efforts: a consequence of its ability to reduce the vectorial capacity of mosquitoes [11,12]. This so called “pathogen-blocking” phenotype is one of many ways in which *Wolbachia* confers a fitness advantage to its host, ensuring maintenance in an insect population. Despite successes of *Wolbachia-*mediated control program trial releases [13,14,15], the mechanisms behind pathogen-blocking are poorly understood. As viruses and *Wolbachia* are both intracellular residents of eukaryotic cells, they both rely upon many host structures and processes to complete their life cycle. Here, we discuss the requirements for virus and *Wolbachia* infection and how they overlap or interfere with each other. Lastly, we unite the cellular determinants for intracellular infection with the current understanding of *Wolbachia*-mediated pathogen-blocking so as to identify promising future directions for understanding what is emerging as a key tool in vector control across the globe.

## 2. Leveraging *Wolbachia* Infections to Inhibit Viral Replication

*Wolbachia* alters the structure and physiology of its host to promote infection. The alteration of host physiology takes many forms, including reproductive manipulations of the host and providing benefits to the host (e.g., protection against pathogens). Despite the fact that these phenotypes are often described as either parasitic or mutualistic, they both confer a relative fitness advantage to infected members of the population [16,17,18]. Additionally, many *Wolbachia* strains straddle the mutualism-parasitism continuum whereby they simultaneously hijack reproduction while conferring direct benefits to the host [12]. It is this combination of reproductive manipulations coupled with beneficial phenotypes such as the protection against pathogens that has made *Wolbachia* such a promising tool for the control of vector borne disease.

### 2.1. Wolbachia-Induced Reproductive Manipulations as Natural Drive Mechanisms

Across Wolbachia, there are four described reproductive manipulations. Three of these skew sex ratios in favor of infected females: the induction of parthenogenesis [19,20], the feminization of genetic males [21], and male-killing [22]. The final reproductive phenotype—cytoplasmic incompatibility (CI)—does not skew sex ratios, but instead creates sperm-egg incompatibilities [23]. Males infected with a CI-inducing Wolbachia strain produce modified sperm that result in early embryonic arrest and mortality, unless “rescued” by the egg from a female with a genetically compatible Wolbachia strain [24,25,26,27,28,29]. Wolbachia-mediated vector control efforts hinge on effective CI. The earliest control program that leveraged Wolbachia-mediated CI entailed releasing large numbers of Wolbachia-infected Culex pipiens male mosquitoes [30]. These male mosquitoes were effectively sterile due to their crossing incompatibility with the local population, and eradication was achieved in three months [30]. Other control programs take advantage of the fact that you can drive traits through a population by releasing CI-Wolbachia infected females (population transformation) instead of CI-Wolbachia infected males (population suppression).

The virulent Wolbachia strain wMelPop was an early contender for vector control programs due to its ability to both induce CI and shorten the lifespan of the host [31]. In the context of vector control, shorter-lived mosquitoes are less likely to take multiple blood meals, thus reducing the opportunity to transmit pathogens acquired in an earlier blood meal. To ensure the success of Wolbachia-transfection, the wMelPop strain was serially passaged in mosquito cell lines for three years before transferring to whole mosquitoes [32]. Despite significant adaptation to the non-native host, the virulence phenotype of wMelPop-CLA was too severe, rendering hosts less competitive than the uninfected members of the population, making it difficult to drive and maintain the symbiont in a population [33]. Current efforts are now mainly focused on using the wMel strain that induces CI and inhibits viral replication without causing severe fitness reductions in the mosquito. wMel infected females are released into populations of mosquitoes naïve to Wolbachia, and CI results in the spread of Wolbachia and the infected matriline [16,17,34,35]. The result is a population of mosquitoes fixed for Wolbachia infection with increased resistance to, and decreased transmission of, viral pathogens [13,15,34,36,37]. A thorough understanding of the mechanisms behind pathogen-blocking will greatly benefit the implementation and maintenance of Wolbachia-based control measures [28,29,38].

### 2.2. Wolbachia Modifies the Host Intracellular Environment

Wolbachia is dependent upon a number of host factors to ensure replication, transmission, and modification of the host. While infection of the developing oocyte is essential for maternal transmission and induction of reproductive phenotypes, Wolbachia infects an array of other tissue and cell types in the host [39,40]. As a result, Wolbachia infection has drastic effects on host physiology, with numerous differences reported between comparisons of infected and uninfected individuals of the same genetic background. These differences manifest at the level of the cell, host, and population, and include effects on gene expression [41,42,43,44,45], macromolecule availability [46], fecundity [31,47,48,49], behavior [50], and even speciation [51,52].

Wolbachia’s effects on host physiology are likely a result of both direct targeting of host processes by Wolbachia, and indirectly, as a consequence of Wolbachia’s residence within host cells. For example, Wolbachia has an intimate relationship with host-derived membranes. Wolbachia resides within golgi-derived vesicles [53], and relies upon endoplasmic reticulum associated protein degradation to maintain titer within host cells [54]. As a result, Wolbachia’s presence significantly alters the morphology of these intracellular membranes [54]. Several studies revealed that Wolbachia has an effect on cholesterol and lipid metabolism and localization [46,55,56], agreeing with Wolbachia’s dependencies on host-membranes. Similarly, Wolbachia has a close relationship with the cytoskeleton, and uses host microtubules and actin to facilitate localization [57,58,59]. There are several described effector proteins that Wolbachia secretes via the Type IV Secretion System (T4SS), that directly target host processes so as to maintain infection [58,60,61,62]. One of these effector proteins, WalE1, is an actin bundler [58].

**Figure 1:**
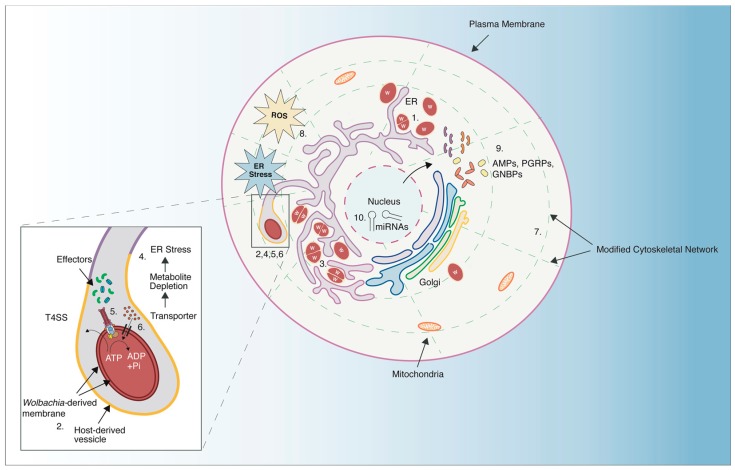
*Wolbachia* modifies the intracellular environment of the host. 1. *Wolbachia* typically exhibits a perinuclear localization, and closely associates with host derived-membranes (*Wolbachia* are marked with a “W”). *Wolbachia* associates with the ER in particular, which results in atypical ER morphologies, including expansion and swelling; 2. *Wolbachia* are enclosed by three distinct membranes: a host derived vesicle (likely of Golgi-origin), and two *Wolbachia* derived membranes (inset); 3. During *Wolbachia* replication, daughter cells temporarily share the host-derived membrane, which later abscises; 4. *Wolbachia* have been observed fused to the ER, and having a direct connection to the ER lumen (inset), likely facilitating the exchange of proteins or other metabolites; 5. The Type Four Secretion System (T4SS) allows *Wolbachia* to export effector proteins directly to the host (inset); 6. The *Wolbachia* genome also encodes a number of transporters that likely facilitate uptake of nutrients from the host; 7. In addition to associating with intracellular membranes, *Wolbachia* is known to alter the host cytoskeleton (depicted as green dashed lines); 8. The presence of *Wolbachia* results in the production of reactive oxygen species (ROS), contributing to cellular stress; 9. Lastly, *Wolbachia* is often associated with upregulation of immune-related genes and pathways including antimicrobial peptides (AMPs), Gram-negative binding proteins (GNBPs), peptidoglycan recognition proteins (PGRPs), and miRNAs (10).

In addition to altering intracellular morphology, the presence of Wolbachia induces changes in host gene expression. Notable differences in gene expression have been reported for antioxidant processes [43,63], metabolism [42,45], immune responses [41,42,43,44], and miRNAs [64,65,66], amongst others. Wolbachia’s surface protein alone seems to be sufficient for eliciting a transcriptional response by the host [67]. Furthermore, there are multiple lines of evidence that Wolbachia infection alters epigenetic patterning of host genomes [68,69,70]. Wolbachia is reliant upon the host for nutrients, both as a result of obligate intracellularity, and Wolbachia’s relatively reduced genome [71]. Indeed, amino acids seem to be the subject of competition between Wolbachia and host [72]. Other research suggests that Wolbachia is providing a benefit to the host in the form of metabolic provisioning [73,74]. Regardless of whether or not Wolbachia is providing a cost or benefit to the host, and how that manifests under different environmental conditions, it is clear that Wolbachia’s presence results in an intracellular environment that is significantly deviated from normal. Because Wolbachia modifies the host environment to favor its own reproduction and transmission, it is perhaps not surprising that this now altered environment is less optimal for incoming viruses (Figure 1).

### 2.3. Wolbachia as a Protective Mutualist

Historically, *Wolbachia* was considered a parasite in insects, due to the aforementioned reproductive manipulations and perturbations of host physiology. However, the discovery that the *Wolbachia* strain infecting *Drosophila melanogaster* (*w*Mel) protects its host against native *Drosophila* viruses brought into question the nature of the host-*Wolbachia* relationship—this was the first evidence of *Wolbachia* providing a direct benefit to an arthropod host [12]. Flies infected with *Wolbachia* survived longer when challenged with virus, and viruses replicated to lower titers as compared to *Wolbachia*-free flies [12]. Viral protection was initially shown for native *Drosophila* RNA viruses including *Drosophila* C virus (DCV), flock house virus (FHV), and Nora virus [12]. Additional research confirmed that this anti-viral protection is not unique to *Drosophila* viruses, but strains of *Wolbachia* native to *Drosophila* inhibit viral replication of an array of arboviruses including dengue virus (DENV) [75], sindbis virus (SINV) [76], West Nile virus (WNV) [77], Semliki forest virus (SFV) [78], and yellow fever virus (YFV) [79]. When transfected into *Aedes* mosquitoes, *w*Mel protects against infection with chikungunya virus (CHIKV), DENV, and Zika virus (ZIKV) [11,13,14,34,80,81,82,83]. In contrast to providing protection against RNA viruses, *w*Mel provided no protection against the DNA virus—Insect Iridescent Virus 6 (IIV-6) [12]. Similarly, field populations of the African armyworm *Spodoptera exempta* (Lepidoptera: *Noctuidae*), naturally infected with *Wolbachia* strain *w*Exel, enhanced host susceptibility to infection with an endemic baculovirus, Spodoptera exempta nucleopolyhedrovirus (SpexNPV), also a DNA virus [84].

After the discovery that *w*Mel protects native and non-native hosts (such as *Aedes aegypti*) against RNA virus infection, myriad *Wolbachia* strains have been tested for pathogen-blocking abilities. Indeed, pathogen-blocking has been confirmed for a number of *Wolbachia* strains, including those from phylogenetically distant clades of *Wolbachia* (referred to as supergroups) and those that naturally infect diverse insect hosts. Within the set of strains that are native to *Drosophila* spp., there is a huge amount of variation in the extent to which hosts are protected from viruses [9,85,86,87]. Strong pathogen-blocking abilities have also been reported for *Wolbachia* strains native to mosquitos (e.g., *w*AlbB, *w*AlbA, *w*Pip) [77,88,89] and a strain native to a leafhopper, *w*Stri [88]. While the coupling of pathogen protection and reproductive manipulations brought *Wolbachia* to the forefront of vector control efforts, even strains that do not induce reproductive modifications can provide strong antiviral protection (e.g., *w*Au) and there is the possibility of maintaining co-infections of different *Wolbachia* strains to “customize” the final pathogen-blocking and reproductive phenotypes [90]. It seems that the ability of *Wolbachia* to protect insects against RNA virus infection is relatively widespread, and likely stems from the ways in which *Wolbachia* establishes within a host.

## 3. The Cellular Context of RNA Virus Infection

**Figure 2:**
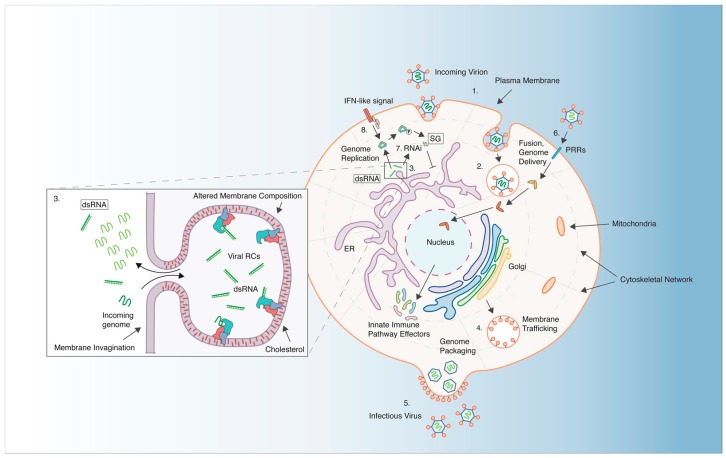
Overview of RNA virus replication in an arthropod cell. 1. Incoming virus particle enters the cell following receptor-mediated endocytosis; 2. Viral genome is delivered into the cytoplasm after the internalized virion escapes the endosome, either by pore-formation or after undergoing fusion with the endosomal membrane; 3. Genome replication occurs inside cytoplasmic virus cores (dsRNA viruses) or within modified membrane-associated structures (see inset) containing virus-encoded replication complexes (RCs). Double-stranded viral RNA (dsRNA) is synthesized as a replication intermediate; 4. After synthesis of viral structural proteins, some are trafficked to the plasma membrane while core proteins encapsulate newly synthesized viral RNA to form cytoplasmic cores; 5. Some viruses obtain their envelope at the plasma membrane before exiting from the cell while others exit following lysis. Presence of virus in the cell also elicits different antiviral responses; 6. Recognition of viral proteins by pattern recognition receptors (PRRs) triggers innate immune pathways, activating transcription factors that induce expression of effectors and antiviral factors; 7. Viral dsRNA triggers RNAi pathways that also aid in viral inhibition; 8. Although poorly understood in arthropods, extracellular interferon-like signaling and the presence of intracellular dsRNA might cause activation of PKR orthologues PERK or GCN2, leading to eIF2α phosphorylation and subsequent stress granule (SG) assembly. Such an event might lead to repression of viral translation and genome replication (inset). Changes in membrane composition and structure is common during RNA virus replication in the cell. Many RNA viruses require the presence of cholesterol (shown in red) or other lipids in the membrane to allow proper localization and functioning of their replication complexes.

Reports of viral inhibition in insects, as mediated by *Wolbachia*, have been restricted to viruses with either positive-sense (+) ssRNA genomes, or double-stranded (dsRNA) genomes [12]. Reports of *Wolbachia*-mediated inhibition of negative sense (−) ssRNA viruses are sparse. Studies analyzing bunyavirus replication indicate at most a mild effect on virus production [77,91]. The absence of support for the ability of *Wolbachia* to protect its host against DNA virus infections could, in part, be due to the absence of well-characterized DNA virus model systems. However, existing data suggest that many *Wolbachia*-host combinations are broadly antiviral against RNA viruses from diverse families [12,81,83]. This suggests the possibility of a common underlying mechanism that might target shared aspects of (+) ssRNA and dsRNA viral life cycles. To this end, we have chosen to outline features of RNA viral life cycles that may be targeted to elicit an antiviral response by *Wolbachia* and its insect host (Figure 2).

### 3.1. Requirements for Virus Entry and Intracellular Localization

At the cellular level, the first objective of a virus is to attach to and enter a host cell. Notably, Wolbachia-mediated pathogen-blocking has been observed against members of both enveloped (Togaviridae, Flaviviridae) and non-enveloped (Dicistroviridae, Nodaviridae, Reoviridae) RNA virus families. Enveloped viruses typically trigger membrane fusion, often through a low pH-mediated conformational change in fusion proteins on the viral surface [92]. This helps the virus cross host membrane barriers to achieve nucleocapsid delivery into the cytoplasm. In contrast, attachment and entry of non-enveloped arboviruses is poorly understood. Given the lack of fusion proteins, these virions are instead thought to trigger and undergo membrane penetration using “penetration proteins” that permeabilize cellular membranes [93,94]. At present, there are no data on whether or not Wolbachia directly interferes with virion attachment and entry. However, several studies report perturbations in cellular lipid levels in the presence of Wolbachia [46,55], which may affect receptor binding and attachment, virion internalization, or virion replication [95,96,97,98].

Following entry into the cell, virus particles are trafficked through the cytoplasm. This occurs while the virus or subviral virion remains as a cargo within endosomes, which are ferried across the cytosol with the aid of cytoskeletal elements, including microtubules and associated motor components [99,100,101,102]. In parallel, cellular cytoskeletal elements, including actin and microtubules, are both considered integral to Wolbachia’s obligate intracellular lifestyle [57,59]. Wolbachia encoded factors have been demonstrated to associate with and modify the function of actin filaments [58]. A host cell harboring a pre-existing Wolbachia infection could therefore exhibit alterations in vesicular trafficking that compromise the delivery of different types of endosomal cargo, including viruses [55].

### 3.2. Requirements for Viral Genome Replication

That pathogen-blocking is specific to a subset of RNA viruses, suggests the possibility of Wolbachia-mediated antiviral effects occurring specifically at a stage of the viral life cycle that is unique to (+) ssRNA and dsRNA viruses. RNA virus genome replication typically occurs in the cytosol within distinct membrane associated structures that house viral replication complexes (RCs). Such virus-induced membrane rearrangements occur in the form of invaginations at specific membranes associated with the ER (Flaviviridae) [103,104], plasma membranes maturing into endosomal and lysomal membranes (Togaviridae) [105,106], double-membrane bound cytoplasmic vesicles (DMVs) (Flaviridae, Picornaviridae) [107] or, in some cases, on outer membranes such as the mitochondria or plasma membrane (Nodaviridae) [108,109]. As is the case with entry via endosomes, viral RCs rely on host cytoskeletal elements for proper trafficking and recruitment of host and viral components to sites of genome replication, as well as for shuttling viral mRNA to sites of viral gene expression [100]. Members of the flavivirus family encode RC proteins that interact with cytoskeletal components and treatment of infected cells with cytoskeleton-disrupting drugs results in an inhibition of viral RNA synthesis [110]. Similarly, in alphaviruses, expression of the host gene vimentin (an intermediate filament protein) is upregulated following CHIKV infection in human muscle cells [99]. Proteomic studies have revealed the role of vimentin in anchoring CHIKV RCs in the cell through interactions mediated by the viral nonstructural protein 3 (nsP3). In other alphaviruses, including SFV and SINV, immuno-precipitation of RC components results in co-precipitation of actin, tubulin, and myosin, which further indicates that the interaction of viral proteins with the cytoskeletal framework is required for the assembly and/or functioning of viral RCs [111].

Additionally, viruses rely on and coopt cholesterol and fatty acid biosynthesis pathways to alter the membrane composition of RCs [112,113]. Combinatorial pharmacological disruption of components of the cholesterol biosynthesis pathway has established the importance of sterol-derived cholesterol biosynthesis during DENV replication, independent of viral entry and egress from the cell [114]. Virus-encoded proteins such as DENV NS3 have also been implicated in the recruitment of fatty acid synthase to establish viral RCs in human Huh7.5 cells [115]. This requirement for cholesterol is also seen in the insect vector: in mosquito cells DENV was inhibited by treatments with an intracellular cholesterol transport inhibitor [55]. WNV upregulates cholesterol biosynthesis while facilitating redistribution of cellular cholesterol and 3-hydroxy-methyglutaryl-CoA reductase (HMGCR, a cholesterol-synthesizing enzyme) to sites of virus-induced membrane platforms [116]. Such membrane rearrangements have been previously reported to involve proliferation of internal membrane structures with the help of WNV proteins NS4A-NS4B, and host enzymes involved in lipid synthesis [117]. Similar to DENV, pharmacological inhibition of cholesterol biosynthesis and RNAi-mediated depletion of HMGCR was shown to inhibit expression of WNV replicon [116]. Taken together, it is evident that the arrangement, composition, and trafficking of internal membranes in the cell is critical for successful viral replication.

### 3.3. Hijacking the Host Translational Machinery

Although viruses exhibit remarkable diversity in the size and complexity of their genome and encoded proteins, they all share one common aspect: absolute dependence on the translational machinery of their host, a prerequisite for viral gene expression. Arboviruses are no exception to this rule. Following the onset of infection, viral genome replication occurs in concert with the expression of viral replicase proteins and involves sequential events of cap-dependent/independent translation initiation, elongation, and termination [118,119,120]. Following the eukaryotic mRNA model, many arboviruses employ the closed-loop model to improve translation efficiency along with engineering the shut-off of cellular cap-dependent translation to reprogram the host cell into expressing viral proteins [121].

Countermeasures employed by the host cell include metabolite repression (such as the depletion of amino acids) and induced stress that together form a part of the cellular response to viral takeover [121]. Protein Kinase R (PKR) mediated phosphorylation of eukaryotic initiation factor 2 alpha (eIF2α) leads to formation of stress-granules and RNA-processing P-bodies that together cause translational arrest of the virus [122]. In mammalian cells, viruses overcome this translational repression either by directly inhibiting PKR activity, or by sequestering stress granule native proteins to viral polysomes to remove an existing translational block while carrying out robust eIF2α-independent translation of certain “late” viral transcripts, a phenomenon observed during infection across alphaviruses including CHIKV, SINV and SFV and hepatitis C virus (HCV, Flaviviridae) [123,124,125,126,127,128]. In each case, viral proteins function directly to counter the host antiviral response. Alphavirus subgenomic transcripts contain a 5′ translation-enhancer element that aids in overcoming the host-induced translational repression. However, this 5′ translation-enhancer element appears to be non-essential in arthropod cells [129]. Additionally, arthropods do not encode a PKR orthologue, though they do have orthologues of general control nondepressible 2 (GCN2) and PKR-like endoplasmic reticulum kinase (PERK), which may act in manipulating eIF2α to play a role during viral infection [130]. Notably, arthropod cells harboring Wolbachia share similar characteristics as cells infected with viruses. Wolbachia lacks essential amino acid biosynthesis genes and is therefore thought to scavenge amino acids from the host cell, and Wolbachia infection is also associated with the induction of cellular stress conditions [55,71,131]. Therefore, in Wolbachia-infected cells this pre-existing “stress” condition may serve to limit viral translation and hence inhibit replication.

### 3.4. Genome Packaging and Exit from the Cell

Packaging the end-products of viral replication involves both host and viral proteins, as well as *cis-*acting elements in the viral genome [132]. Examples include the conserved packaging sequences in the genomes of alphaviruses (*Togaviridae*) that confer packaging specificity [133]. For flaviviruses, virus replicons seem particular to their own structural proteins (C/prM/E), suggesting the importance of homology between the viral genome and flavivirus C proteins for genome packaging and the formation of infectious virus-like-particles [134]. Additionally, recent evidence has implicated N6 methyl-adenosine (m^6^A) methylation of ZIKV genomes in regulating infectious virus output in mammalian cells [135,136]. It remains to be seen whether or not other chemical modifications of viral RNA such as 5-methyl cytosine (m^5^C) methylation have similar effects on viral assembly, and what the consequences of viral genome methylation are in arthropod cells.

Following viral assembly, exit from the cell requires the assistance of cellular trafficking machinery akin to those required for virus entry. Both the cytoskeleton and associations between viral proteins and cellular membranes are key determinants of viral exit [137,138]. Once again, this leaves open the question of whether or not *Wolbachia*-induced modifications of the actin cytoskeleton or perturbations of cellular cholesterol contribute to virus dissemination through blocking of viruses exiting from the cell.

## 4. Insect Responses to Viral Infection

Arbovirus infection in vertebrates is followed by an immediate innate immune response that leads to a subsequent system-wide adaptive response, specific to the invading virus [139,140,141,142]. This adaptive response forms a part of the organism’s immunological memory that combats re-exposure to pathogens. In contrast, arthropods have been traditionally considered to lack a similar adaptive response, but instead possess an array of interconnected signaling pathways that result in an immediate systemic immune response to virus infection [143]. However, there is recent evidence for an RNAi-based adaptive antiviral response in *Drosophila*, potentially leading to immunological memory [144]. In the following sections, we discuss challenges that viruses face in arthropods with regard to host immunity, as well as mechanisms of immune evasion evolved by viruses.

### 4.1. Evasion of Host RNA-Mediated Gene Silencing

Throughout the course of infection, viruses must evade or overcome anti-viral responses mounted by the host. RNA-interference machinery is one such anti-viral mechanism, triggered by the presence of intracellular double-stranded RNA (dsRNA) that leads to the production of small RNA molecules (e.g., short-interfering (siRNA), micro-(miRNA) and Piwi-interacting (piRNA)) that regulate the expression of cognate RNA targets [145]. RNA viruses have developed an array of tools to avoid targeting or degradation by RNAi pathways. The viral B2 gene product of the insect pathogen, FHV, binds to and neutralizes the antiviral effect of siRNAs in D. melanogaster [146]. In other cases, arboviruses, such as DENV, sequester replication-intermediate dsRNAs into membrane-associated structures, as seen in C6/36 Aedes albopictus cells [147]. Other strategies include the use of superfluous DENV vRNA as a “decoy,” while others involve WNV and SFV genomes harboring siRNA “hotspots” carrying point mutations that make them less efficient at silencing viral gene expression [148,149].

### 4.2. Innate Immune Responses to Viral Infection

In addition to RNAi, several signaling pathways play important antiviral roles in arthropods through regulation of protein-coding gene expression. Functional characterization of such pathogen-inducible pathways, including Toll, Immune deficiency (Imd) and Janus Kinase/Signal Transducer and Activator of Transcription JAK/STAT, have primarily been carried out in the D. melanogaster model but have also been reported in other insects including mosquitoes, suggesting functional conservation. Toll and Imd comprise NFκB-dependent pathways that are involved in the cell’s response to virus infection. The Toll pathway is homologous to the Toll-like receptor (TLR) signaling pathway found in vertebrates, also known to function in the context of antibacterial (gram-positive) and antifungal defense [150,151]. Activation of Toll and Imd occurs following recognition of virus components as pathogen-associated molecular patterns (PAMPs) by pattern recognition receptors (PRRs). This information is then relayed into the cell via signaling pathways that result in transcriptional activation of host genes. In flies, the antiviral role of the Toll pathway has been demonstrated during native Drosophila X Virus (DXV: Birnaviridae) infection, shown by reduced survival of flies lacking the Toll-pathway transcriptional activator Dorsal-related immune factor (Dif) [151]. Additionally, this pathway is reported to be functionally important during infection by different DENV serotypes in Ae. aegypti mosquitoes [131,152]. RNAi-mediated silencing of cactus, a negative regulator of the Toll-pathway, resulted in reduced DENV titers, while silencing the pathway component MyD88 lead to increased virus titers in the insect [152].

Imd, the other canonical NFκB-dependent pathway, functions in the context of bacterial pathogens (gram-negative, activated by DAP-type bacterial peptidoglycans) and viruses [153,154]. PRR recognition in this case leads to subsequent intracellular signaling that allows translocation of the NFκB transcription factor Relish to translocate into the nucleus and activate expression of effector molecules [155,156]. Akin to Toll, the functional loss of Imd pathway components, including Relish, has been demonstrated to increase cricket paralysis virus (CrPV: Dicistroviridae) load and virus-induced mortality in flies [154]. Additionally, Imd pathway components and Imd-regulated antimicrobial peptides have been demonstrated to function as antivirals in the context of alphavirus infection [157]. Priming the Imd pathway by pretreating mosquito cells with heat-inactivated, gram-negative E. coli also leads to reduced SFV infection, suggesting its role as an antiviral during early SFV infection [158]. Viral genome replication, launched from a genomic-encoded SINV-replicon in flies, is increased in Imd pathway mutants, while RNAi-mediated depletion of the Imd pathway effector Diptericin B (DptB) leads to an increase in viral genome replication and titer [157,159].

The last pathway, JAK/STAT, is activated by viral infection and cross-talks considerably with the RNAi pathway. Many viruses such as DCV, FHV, and DXV induce expression of canonical JAK/STAT genes and inactivation of the Janus kinase Hopscotch (Hop) leads to increased DCV load in flies [160,161]. The siRNA pathway component Dicer-2 is known to activate the transcription of an antiviral gene—vago—previously reported to be important in conferring resistance to DCV in flies [162]. However, its antiviral role is independent of the host’s siRNA pathway. Instead, Vago is secreted outside the cell where it binds to and activates the JAK/STAT signaling pathway. This cytokine-like function of Vago and its involvement in activation of the JAK/STAT pathway is akin to how this pathway functions in vertebrates, where it is involved in canonical antiviral signaling mediated by interferons [163]. In flies, where its role was initially characterized during development, JAK/STAT can also be triggered in a canonical, vago-independent manner which involves PRR-binding to distinct receptors on the cell surface [164,165]. In Ae. aegypti, the JAK/STAT pathway is activated upon DENV infection, while RNAi-mediated silencing of pathway components such as the receptor, Domeless and the Hop kinase, leads to increased viral loads [166]. In contrast, WNV infection in Culex quinquefasciatus activates the Dicer-2-mediated JAK/STAT pathway by inducing the expression of CxVago [167]. Loss of CxVago is accompanied by elevated WNV replication in mosquito cells, suggesting its role in restricting viral replication in cells [167].

It should be noted that in contrast to viral evasion strategies against insect RNAi machinery, none have been described to act against Toll, Imd, or JAK/STAT in insects. Still, multiple flavivirus proteins such as the WNV envelope protein, DENV nonstructural proteins, and NS5 proteins of WNV, DENV, Japanese encephalitis virus (JEV: Flaviviridae), and tick-borne encephalitis virus have been shown to inhibit cytokine production, signaling, and NFκB activation in vertebrate cells [168,169]. Similar strategies may exist in arthropod cells for viruses to escape pattern recognition and subsequent activation of Toll, Imd, or JAK/STAT.

## 5. Cellular and Molecular Signatures of Pathogen-Blocking

**Figure 3:**
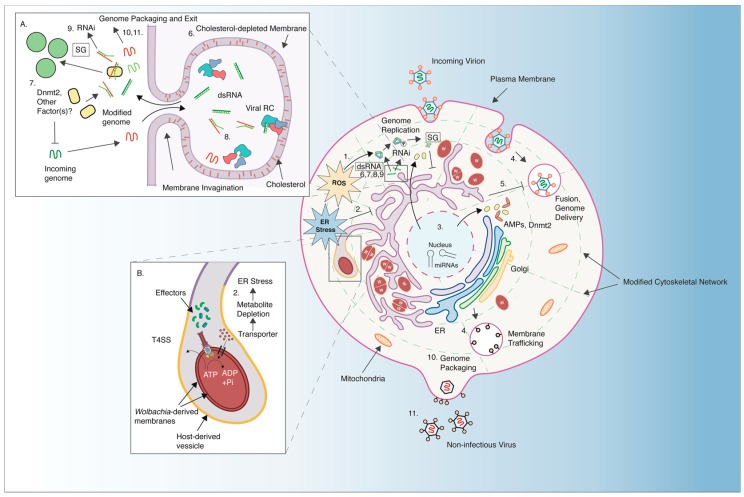
*Wolbachia*-mediated virus inhibition in arthropods. *Wolbachia*-mediated virus inhibition is likely a cumulative effect arising from multiple roadblocks at different stages of virus life cycle. 1. Reactive oxygen species (ROS) are produced in a *Wolbachia-*infected cell that might lead to stress granule (SG) assembly (inset (**A**)); 2. Moreover, competition for intracellular cholesterol and amino acids (inset (**B**)) between virus and *Wolbachia* may lead to metabolite depletion, giving rise to ER stress in the cell and vice versa; 3. Presence of *Wolbachia* can also trigger expression of host miRNAs, antimicrobial peptides (AMPs) and genes associated with antiviral immunity (e.g., Dnmt2, Toll pathway genes); 4. Modulation of the cellular cytoskeletal network (depicted as green dashed lines) by *Wolbachia* might disrupt vesicular trafficking, thus affecting virus entry and/or exit steps; 5. Cholesterol depletion in membranes might affect viral genome uncoating and delivery into the cytoplasm (inset (**A**)); 6. Additionally, lack of cholesterol in membranes (shown in red) might disrupt assembly and functionality of viral replication complexes (RCs), abrogating viral genome replication; 7. Post-transcriptional modification (PTM) of viral RNA (vRNA) by the host RNA methyltransferase Dnmt2 might allow viral RNA trafficking to SGs, leading to inhibition of viral genome replication; 8. PTM of vRNA on its own can also compromise its ability to be replicated, leading to reduced viral protein synthesis; 9. PTM modified and/or Dnmt2-bound vRNA might also lead to RNAi-mediated virus inhibition; 10. Modified vRNA may also cause improper packaging into virions; 11. Defects in virion structure and/or modified nature of the encapsidated vRNA might result in the production of virus particles that are incapable of initiating a fresh round of replication in other cells, limiting virus spread from cell-to-cell.

While it is exciting to speculate a conserved, unified mechanism of antiviral resistance, it is nonetheless difficult to disentangle mechanistic information reported in different arthropod models harboring different *Wolbachia* strains. To circumvent this issue, our discussion of pathogen-blocking treats *Wolbachia*-colonized arthropod cells as a singular entity, with the bacterium modifying the existing cellular environment in ways that are refractory to RNA virus infection, consistent with a model of blocking occurring early in virus infection [76,78]. As the currently proposed mechanisms for pathogen-blocking have not been thoroughly explored across different *Wolbachia*-host-virus combinations, it is difficult to say how well one study translates to a different *Wolbachia*-host-virus system. However, certain pathogen-blocking phenotypes have been repeatedly reported over the span of the last decade, across myriad *Wolbachia*-host associations. These include the induction of cellular stress (e.g., oxidative and ER stress), and perturbation of cellular cholesterol levels [55,56,131,170,171]. In the next few sections, we discuss current evidence that support the idea of *Wolbachia-*induced stress as a fundamental component of the cellular antiviral response (Figure 3).

### 5.1. Presence of Wolbachia Induces of Cellular Stress

Early proteomic experiments revealed that association of native Wolbachia in Aa23 Ae. albopictus cells is accompanied by increased expression of the host anti-oxidant proteins superoxide dismutase (homologous to D. melanogaster cytoplasmic CuZnSOD), peroxiredoxin and glutathione peroxidase, and the Wolbachia proteins superoxide dismutase and bacterioferritin [63]. While production of anti-oxidant proteins implies imbalance of redox homeostasis in the cell, identification of increased superoxide dismutase levels suggested the source of oxidative stress as being cytoplasmic, and not mitochondrial or extracellular in origin [63]. Indeed, while flow cytometric analysis indicated elevated cytoplasmic reactive oxygen species (ROS) levels among a fraction of Wolbachia-colonized cells, microscopic analysis of such cells suggested co-localization of ROS with DNA in the cytosol, indicating ROS production within intracellular cytoplasmic compartments housing the bacterium. This agreed with Wolbachia upregulating expression of its own anti-oxidant proteins, given that Wolbachia density is sensitive to ROS [171]. Additionally, proteomic analyses of wMelPop infected Ae. albopictus cells and wMel-infected mosquito midguts indicated stress-related differential gene expression, as inferred by upregulation of ER proteins involved in protein folding and glycosylation, suggesting the onset of cellular unfolded protein response [55]. Collectively, these data support the idea of Wolbachia acting as a source of stress in the arthropod cell.

While unusually elevated ROS levels lead to disruption of biological macromolecules such as proteins, nucleic acids, and lipids, at physiological levels, ROS are known to act as signaling molecules to activate a wide range of signaling pathways, which include the extracellular signal-regulated kinase (ERK) pathway [172,173,174]. Capable of being activated by external stimuli and intracellular events, MEK/ERK signaling is a conserved MAPK pathway involving a series of phosphorylation events that regulate gene expression [175]. Using RNAi screens in mosquito (Aag2) and Drosophila (DL1, KC167) cells, components of the ERK pathway have been demonstrated to be critically important in mediating protection against RNA viruses such as SINV and DCV [176]. ERK signaling is active in the Drosophila gut, and can be induced by members of the insect gut microbiota to act in an antiviral capacity [177]. Remarkably, oral treatment with an inhibitor against the ERK pathway component MEK allow otherwise non-permissive RNA viruses to escape the intestinal barrier to infect gut epithelial cells [177]. These observations led researchers to ask whether or not Wolbachia-induced ROS production triggers signaling via the ERK pathway to mediate antiviral protection. Indeed, wMel induces ERK signaling following ROS production, demonstrated by an increase in phospho-ERK in Wolbachia-infected cells [178]. Additionally, loss-of-function ERK mutants succumb to DCV oral infection faster than wild-type flies, without an accompanying loss in Wolbachia density.

### 5.2. Roles of Cholesterol and Lipid Imbalance in Virus Inhibition

The role of disrupted cellular cholesterol and lipid homeostasis in virus resistance has been reported across a range of Wolbachia-host associations [46,55,56,88]. That such imbalance exists in this cellular context is not surprising given that certain conditions of stress (e.g., ER stress and activation of unfolded protein response) are linked to lipid biosynthesis [55,179,180]. Proteomes of wMel- and wMelPop-infected Ae. aegypti cells, and wMel-infected mosquito midguts, revealed significant changes in expression of genes associated with lipid metabolism [46,55]. Consistent with these results, there were elevated levels of esterified cholesterol in the presence Wolbachia, along with a concomitant reduction in free cholesterol. Artificial labeling of cholesterol in Wolbachia-infected cells revealed the presence of localized lipid droplets. Similar results were obtained from a separate study comparing the effects of wMel and wMelPop infections in Drosophila on cholesterol competition between the bacterium and the host [56]. Additionally, lipidome analysis of wMel and wMelPop infected Ae. albopictus cells showed decreases in sphingolipid levels, particularly ceramides, in the presence of Wolbachia [46]. The extent of ceramide depletion positively correlated with Wolbachia density. In contrast, there was differential regulation of phospholipids, with increases in phosphatidylcholine and phosphatidylinositol, and a decrease in phosphatidylserine levels.

On the other hand, RNA viruses, especially enveloped flaviviruses and alphaviruses, rely on cellular cholesterol at multiple stages of the viral life cycle, including entry, replication, virion assembly, and exit. Presence of cholesterol in the DENV envelope is critical for initiating virion uncoating at the start of infection, while the alphaviruses SINV and SFV rely on cholesterol to mediate membrane fusion during entry into the host cell [95,96,97]. It is therefore logical to hypothesize that competition for cholesterol occurs in cells during Wolbachia-virus co-infections, with Wolbachia winning out at the end due to its precedence. This hypothesis has been tested in various Wolbachia-host combinations, in the context of three viruses: DENV, ZIKV, and DCV. Indeed, recent evidence suggests an increase in esterified cholesterol levels in Wolbachia-infected mosquito cells that greatly reduces the amount of free cholesterol that is available in the cell, which is required for virus replication. Supplementation of a cholesterol-binding compound that solubilizes said esterified cholesterol to wMelPop infected Ae. aegypti cells leads to a greater than 100-fold increase in DENV genome copies in the presence of Wolbachia, indicating that availability of free cholesterol may be limiting during DENV infection of Wolbachia-colonized cells [55]. However, DENV rescue was not observed in the same wMelPop-infected cells following addition of exogenous cholesterol. This is in contrast to observations made in wStri-infected C710 Ae. albopictus cells, where addition of cholesterol-lipid supplement partially rescues growth of ZIKV, demonstrated by a 10-fold increase in ZIKV genome copies [88]. It should be noted, however, that infection of flaviviruses for example, DENV and JEV has been shown to be highly sensitive to imbalances in cellular cholesterol levels and that although its required for entry, presence of excess cholesterol in cellular membranes inhibit stages of the viral life cycle such as entry and replication [181]. The apparent conflict in outcomes of the two cholesterol supplementation experiments described above could therefore arise as a result of experimental setup, that is, exogenous cholesterol versus cholesterol-lipid supplement, causing differences in cholesterol abundance and distribution within the cell [55,88]. Finally, DCV load and host mortality increased if Wolbachia-infected flies were previously reared on a high cholesterol diet [56]. However, it should be noted that the flies used in this study were maintained in cholesterol-enriched diets over multiple generations, leaving open the possibility that host adaptation might explain the change in the pathogen-blocking phenotype [56].

### 5.3. Pathogen-Blocking Resulting from Competition for Cellular Resources

The genome of the Wolbachia strain wMel contains several predicted amino acid transporters including those for proline, asparate/glutamate and alanine, suggesting the use of amino acids as a primary nutrient source [71]. Interestingly, supplementation of blood meals with single or multiple amino acids rescued fecundity and egg viability defects caused by wMelPop infection in Ae. aegypti [72], suggesting depletion of amino acids by the Wolbachia infection. Viruses also exploit the cellular amino acid pools, relying on host translational machinery to propagate, with the success of CHIKV and DENV in mosquito cells depending on the amino acid composition of the growth media [72,182]. Depletion of the host amino acid pool could lead to translational arrest via eIF2α phosphorylation, which, as discussed earlier, might contribute to the observed blocks in virus replication [183]. Moreover, given that amino acid sufficiency is required for resistance against induced oxidative stress, lack thereof might therefore lead to a loss in the cell’s ability to quickly overcome such a state.

### 5.4. Role of RNA Methyltransferase Dnmt2 in Regulating RNA Virus Infection

The RNA methyltransferase Dnmt2 is a host-encoded gene recently implicated in the control of viruses, and in Wolbachia-mediated viral inhibition [63,76,131,184,185]. Following infection with (+) ssRNA viruses like DCV and Nora virus (Picornaviridae), Dnmt2 loss-of-function mutants accumulated higher viral loads and succumbed to infection faster than the wild-type counterparts [184]. RNAi-mediated depletion of Dnmt2 in D. melanogaster cells resulted in increased SINV replication and improved virion infectivity going into mammalian BHK-21 cells [76]. In the same study, infection with wMel resulted in the elevated expression of Dnmt2, and a decrease in virion infectivity. Wolbachia-infected Dnmt2 loss-of-function mutants and Dnmt2 knock-downs challenged with SINV were no longer resistant to the virus [76]. SINV genomes and sub-genomes have been historically reported to contain m^5^C methylated residues whose role during infection remains undefined [186]. In contrast to the antiviral role of D. melanogaster Dnmt2, expression of its mosquito homolog, AaDnmt2 was downregulated in wMelPop-CLA-infected Ae. aegypti mosquitoes via a Wolbachia-induced host miRNA, which further correlated with inhibition of DENV infection [187].

Distinct forms of RNA methylation (e.g., 5-methylcytosine (m^5^C), N6-methyladenosine (m^6^A) and Pseudouridine (Ψ)) have different functional implications for the control of cellular RNA species [188]. Such modifications of mRNA can affect almost every aspect of form and function, from RNA structure and translation efficiency (m^6^A, m^5^C and Ψ), to genetic recoding (m^5^C and Ψ), to mRNA stability, export and cap-independent translation (m^6^A). Still, we have only recently started to discover the distribution and impact of viral RNA methylation on genome functionality [189]. Nevertheless, recent data have uncovered an overwhelming number of post-transcriptional modifications that suggest a previously unknown and therefore, unappreciated form of RNA virus regulation in eukaryotes [135,189].

It is currently unknown whether Dnmt2′s antiviral function, either independently or in the context of Wolbachia, involves its enzymatic role as a RNA methyltransferase. In the context of its canonical cellular target, (tRNAs) Dnmt2 has been demonstrated to bind to and methylate specific cytosine residues located on the anti-codon loop of tRNA_Asp_ [190]. It is also noteworthy that Dnmt2 binds specifically to internal ribosomal binding sites (IRES) located on the 5′ end of DCV RNA, given the structural similarities between tRNAs and IRES elements [184]. Most RNA virus genomes consist of secondary structures in 5′ and 3′ non-coding regions that act as cis-acting regions to regulate multiple aspects of viral infection, including plus- and minus-strand RNA synthesis [191,192,193,194]. In many cases, disruption of these higher order RNA structures is detrimental to viral genome replication [191,194]. It is possible that Dnmt2 functions by either binding to or altering the methylation of cytosine residues at these regions, or elsewhere in the genome, leading to disruption of RNA structures. These changes could inhibit viral replication by preventing genome cyclization, abrogating translation, or by preventing packaging into virions, in a manner similar to that observed recently in the case of m^6^A-methylated HCV RNA in mammalian cells [135].

In D. melanogaster-derived S2 cells, the induction of oxidative stress is associated with subcellular relocalization of Dnmt2 to stress compartments [195]. Similarly, in mammalian cells under oxidative and ER stress, Dnmt2 localizes to cellular stress granules and P-bodies where it interacts with proteins involved in RNA processing [196]. Moreover, while Dnmt2-mediated methylation of tRNAs is known to increase its stability by protecting against stress-induced degradation in P-bodies, the reverse could be true for RNA virus genomes [195,197]. It should be noted that independent of its enzymatic activity, binding of Dnmt2 to viral RNA could also allow for better recognition by other antiviral pathways in the cell or result in an otherwise perturbed RNA structure. Collectively, these data construct a picture of a Wolbachia-infected cell where the presence of the endosymbiont leads to the induction of oxidative and/or ER stress, allowing cytoplasmic Dnmt2 to methylate or “mark” viral RNA and target it for degradation via P-bodies associated with cellular stress compartments. Alternatively, or in addition to this, methylated viral RNA can be packaged into virions that upon re-infection into naïve mammalian or arthropod cells, fail to establish a productive infection, thus limiting virus dissemination within the host.

It is also possible that Dnmt2′s enzymatic role extends beyond viral RNA to include host RNA and/or DNA. DNA methylation in dipterans such as D. melanogaster, Anopheles gambiae, and Ae. aegypti, is generally considered to be sparse (<0.5–6%), except during embryonic development, in the case of Drosophila [198]. Nevertheless, in the case of wMel-infected D. melanogaster, the presence of Wolbachia increases cytosine methylation in testes two-fold, with germline-specific Dnmt2 overexpression leading to a modest 15% reduction in endosymbiont titer in the reproductive tract [69]. In contrast, wMelPop-CLA causes widespread changes in cytosine methylation patterns of the Ae. aegypti genome, mostly in genes associated with host membranes [68]. However, functional implications of such changes remain unknown, making it difficult to postulate their role in antiviral defense, except that Wolbachia titers may be indirectly regulated by changes in Dnmt2 expression.

### 5.5. Host RNA Interference Pathways and Pathogen-blocking

The RNAi pathway plays an important antiviral role in the context of an infection in arthropods. Studies have therefore been conducted to assess the role of RNAi, specifically the exogenous siRNA pathway, in Wolbachia-mediated pathogen-blocking. Challenging wMel-infected D. melanogaster mutants lacking functional components of the siRNA pathway, Dicer-2 (Dcr-2) and Argonaute-2 (Ago2), with DCV, led to increased survival rates compared to the Wolbachia-cleared mutant counterparts, suggesting the siRNA pathway was not essential for Wolbachia-mediated pathogen-blocking [199]. More recently, wMel-infected and uninfected D. melanogaster cells were challenged with SFV and screened for the production of virus-derived siRNAs (viRNAs) as a proxy to determining RNAi activity. While SFV inhibition was observed in Wolbachia-infected cells, a lack of detecTable 21-nt viRNAs led authors to conclude that the RNAi pathway is not required for Wolbachia-induced pathogen-blocking in Drosophila [78]. However, conflicting evidence has emerged from experiments involving wMel-infected Ae. aegypti Aag2 cells, where targeted depletion of Ago2 resulted in DENV recovery, thus arguing for the importance of this RNAi pathway for DENV control in Wolbachia-infected mosquitoes [200].

Little is known about the effect of the RNAi pathway function on cellular stress responses, other than proper functionality of the host endo-siRNA pathway is required for heat shock-induced stress resistance in D. melanogaster [201]. However, in flies under conditions of stress, proper functioning of the cellular siRNA pathway is hampered due to transient reduction in Dicer-2 activity, which is caused by increased fragmentation of cellular tRNAs [185]. In this scenario, fragmented tRNAs are bound by Dicer-2 only to be processed into small sRNAs that compete with cellular dsRNA, thereby reducing Dicer-2 activity on these substrates. Dnmt2 methylates cellular tRNAs, conferring stability and thus reduces stress-induced fragmentation and restores Dicer-2 function [185,195].

Unlike siRNAs, the biogenesis of other interfering RNAs, such as piRNAs, occurs from negative-sense RNAs and does not require the presence of Dicer or a dsRNA precursor [202]. Interestingly, there is evidence that suggests a difference in small RNA production during (+) ssRNA and (−) ssRNA virus infections in mosquito cells [91]. Notably, the presence of an insect-specific flavivirus (cell-fusing agent virus, CFAV) leads to the production of siRNAs (21 nt, Dicer-2 produced, dsRNA precursor), while piRNAs (26–30 nt, negative-sense RNA precursor) are produced during bunyavirus (Phasi-Charoen like virus, PCLV) infection. Moreover, Wolbachia-mediated inhibition of CFLV, but not PCLV is observed in these cells, further suggesting the importance of an siRNA-based antiviral response and a potential reason underlying Wolbachia’s inability to block (−) ssRNA virus infection [91].

Lastly, recognition of viral dsRNA may be required for Wolbachia-mediated pathogen-blocking in arthropods. As discussed, Wolbachia’s antiviral activity towards RNA viruses excludes those with (−) ssRNA genomes. A recent study showed differences in dsRNA quantities in cells infected with (+) ssRNA, (−) ssRNA and dsRNA viruses [203]. Notably, antibody-dependent recognition of viral dsRNA is sparse in cells harboring (−) ssRNA viruses, whereas appreciable levels of dsRNA were detected during infection with (+) ssRNA and dsRNA viruses. It is possible that a Wolbachia-mediated antiviral effect requires the recognition of viral dsRNA, as observed during infection with (+) ssRNA and dsRNA viruses.

### 5.6. Wolbachia Induces the Expression of Antimicrobial Peptides and Toll Pathway Genes

While the presence of Wolbachia is widely associated with changes in host gene expression, interesting differences arise between native and transinfections of Wolbachia in insects when it comes to the upregulation of innate immune genes. Notably, the transinfection of non-native Wolbachia strains in mosquitoes results in the upregulation of innate immune genes. Such changes are generally absent from native Wolbachia-host associations [75,200,204,205]. In D. melanogaster, physiological consequences of constitutive upregulation of immune genes via overexpression of peptidoglycan recognition protein PGRP-LE are dire, contributing to reduced host lifespan [206]. In this instance, while the host is protected against pathogens there is a trade-off between pathogen protection and longevity. As Wolbachia fitness relies on the fitness of its host, an argument can be made as to why immune activation is lacking from long-term, co-evolved host-bacterial associations [205].

In the case of wMelPop-CLA-transinfected Ae. aegypti mosquitoes, 39% of upregulated genes (78 out of a total 199) exhibit putative immune-related functions, including Cecropin genes CECE and CECD and peptidoglycan recognition protein PGRPS1, which ultimately correlates with reduced filarial infection in the host [204]. wMelPop-CLA infection was also found to greatly reduce average lifespan of the insect, which the authors attributed, in part, to elevated immune gene expression. The virulent wMelPop-CLA upregulated a higher number of immune genes compared to wMel transinfections in Ae. aegypti, [75], although the effect on virus inhibition was not investigated. Genes upregulated in the presence of both Wolbachia strains included previously described AMPs such as cecropins, defensin, diptericin and several Toll pathway genes (PGRP-SA, GNBPB4 and GNBPA1). In line with prior findings, the authors also showed that Wolbachia strains wMel and wMelPop (not the CLA strains) in their native D. melanogaster host do not induce expression of these immune genes [75]. Finally, evidence supporting the importance of stress-induced immune priming comes from a study reporting the role of ROS in activating the Toll pathway and production of AMPs in wAlbB-infected Ae. aegypti [131]. Furthermore, RNAi-mediated depletion of Toll, defensin, and cecropin in these wAlbB-infected mosquitoes led to the rescue of DENV titers when mosquitoes took infected blood meals, suggesting their involvement in Wolbachia-mediated DENV resistance. In contrast, wMelPop-infected D. melanogaster mutants in Spätzle (Toll pathway) and Relish (Imd pathway) do not result in increased DENV loads relative to wild-type after intrathoracic injection with virus [207]. These results might be explained by the observation that both the type of virus and the route of infection determine whether or not an immune pathway is required for controlling the infection [151,208]. Additionally, it should be noted that the data on elevated immune gene expression in native Wolbachia-host associations (e.g., wAlbB in Ae. albopictus or wMel in D. melanogaster) are inconsistent. These disparities may be a result of specific genotype-by-genotype interactions, methods of transcript detection (i.e., microarrays, qRT-PCR, RNA-Seq) or how statistical significance was determined. Indeed, the relative contribution of “immune priming” to the pathogen-blocking phenotype remains a source of debate in the field.

### 5.7. Wolbachia Density Predicts Pathogen-blocking

Across the aforementioned pathogen-blocking studies and Wolbachia-host associations there is consistently a strong positive correlation between Wolbachia density and the extent of the pathogen-blocking phenotype [87,88,209,210]. In a panel of different Drosophila-Wolbachia strain combinations tested for resistance against FHV, the extent of pathogen-blocking was explained by Wolbachia strain rather than the host species; the strains that were stronger pathogen blockers were also those that consistently infected hosts at higher titers [211]. These data also suggest that regulation of Wolbachia density is encoded by Wolbachia. The genetic basis of this density regulation has been best explored in the related D. melanogaster infecting strains of Wolbachia—wMel, wMelCS and wMelPop—infecting at progressively higher density. Genomic comparisons between these three strains found single nucleotide polymorphisms distributed across the genome, as well as the duplication of a short genomic region (~21 kb) [86]. A total of 59 non-synonymous mutations have been identified between wMel and wMelCS, mapping to 55 genes while many fewer such changes exist between wMelCS and wMelPop. Genes that harbor mutations, such as the ankyrin repeat domain containing proteins, might have important functional implications regarding symbiosis as they have long been thought to be involved in host-symbiont interactions [212]. However, more direct evidence is available for the ~21 kb duplicated region, known as Octomom, where higher copy number results in higher Wolbachia density [86]. The region is flanked by direct repeats and encodes eight genes, including several ankyrin-proteins, reverse transcriptases and proteins involved in DNA repair. Copy number variation of the Octomom region is highly dynamic and ranges from 0 to 1 in wMel and wMelCS, and up to 7 in wMelPop. Given that Octomom is involved in density regulation, and density correlates pathogen-blocking, the amount of cellular stress induced by Wolbachia infection may correlate with Octomom copy number.

## 6. Genotype by Genotype Interactions

### 6.1. Variability in the Blocking Phenotype

Despite the fact that many Wolbachia strains protect many hosts against viral replication, the pathogen-blocking phenotype manifests differently depending on the context of the infection. It is clear that there are genotype by genotype by environment (GxGxE) interactions at play in the system. Different Wolbachia strains, host species, viral challenges, and environmental conditions all interact with each other to determine the final phenotype. Given that Wolbachia titer so strongly correlates with the level of pathogen-blocking [86,87], any aspect of the host-symbiont relationship regulating Wolbachia density is likely to have effects on the pathogen-blocking phenotype. Wolbachia based vector control programs often involve re-locating a Wolbachia strain from its native host to an introduced host, which significantly changes the nature of the interaction. Hosts infected with a novel Wolbachia symbiont differentially regulate the expression of many immune pathways [75,213], Wolbachia titers change [214,215], and the relationship is now subject to very different selective pressures [216]. There are a vast number of studies that report on the molecular signatures of pathogen-blocking for different Wolbachia-host-virus combinations, using different infection scenarios, environmental conditions, and techniques. Additional layers of complexity include the sex of individuals used in experiments, (likely an important consideration for a maternally transmitted symbiont, and for hosts species in which only females transmit pathogens), and whether or not cell culture or whole animals were used (also important given that Wolbachia densities in particular tissues correlate with blocking [217]). Here, we compare native and non-native Wolbachia-host associations, so as to better understand how infection context relates to the pathogen-blocking phenotype. Cellular stress would logically manifest differently in native, adapted host-Wolbachia associations (versus those that are not co-adapted), and this may be the reason for discrepancies in the pathogen-blocking phenotype seen across Wolbachia-host associations.

### 6.2. Native Host-Wolbachia Associations

Under conditions of primarily maternal transmission, and long-term association of host and Wolbachia, Wolbachia evolves to increase the fitness of its host [16,215,218,219]. Native associations likely represent a relatively stable relationship. Given that high Wolbachia titers can be costly for the host (e.g., wMelPop [31]), it makes sense that this cost also comes with the benefit of strong protection against viral pathogens that have significant negative fitness consequences for the native host [12]. Across Drosophila, most of the native Wolbachia strains provide relatively strong protection against viruses, which is in agreement with their role as mutualists, providing protection against many Drosophila-specific viruses. Indeed, Drosophila-infecting Wolbachia in their native host have been shown to protect against DCV, Nora virus, FHV, and CrPV, as well as arboviruses such as SINV, SFV, WNV, and Blue Tongue Virus (BTV: Reoviridae) [12,76,77,78,85,220]. These native Wolbachia-host associations seem to be primed for resistance to a wide array of viruses. There are of course exceptions to this trend. For example, the wHa and wNo strains that naturally infect Drosophila simulans do not appear to protect against pathogens [85]. However, it should be noted that these two strains naturally occur in co-infections [221], either with each other or with other Wolbachia strains, so it is possible that this ecological and evolutionary difference has influenced assessment of the pathogen-blocking phenotype when strains are considered individually.

In contrast, there is mixed evidence for pathogen-blocking in many of the Wolbachia strains native to mosquitoes. This may be a result of the types of viruses to which Drosophila and mosquitoes are naturally exposed. While there is evidence that arboviruses do impose a fitness cost on their host [81], it is not as severe as the mortality that DCV and similar viruses cause in Drosophila. wAlbA and wAlbB, Wolbachia strains that naturally infect Ae. albopictus and the native wPolA strain infecting Aedes polynesiensis had no effect on CHIKV and DENV, respectively [89,210]. Further, researchers observed mixed results of wAlbB infection; while wAlbB does not inhibit CHIKV or DENV replication, DENV infection of the salivary glands is affected, resulting in reduced transmission [89]. Similarly, there is mixed evidence for the ability of Wolbachia strains native to Culex mosquitoes to limit pathogens for which Culex is the natural vector, such as WNV. Both low and no pathogen-blocking have been reported for the wPip-Culex-WNV association, and it appears that here too, Wolbachia titer is the determinant of whether or not this phenotype manifests [77,222]. In general, it seems that the native Drosophila-Wolbachia are more likely to strongly block pathogens in their native host than are the native mosquito Wolbachia strains in their native host.

### 6.3. Features of Non-Native Associations

The transfer of Wolbachia to novel hosts is the basis of many ongoing vector control programs, mainly focused on reducing the vector competence of Ae. aegypti, which has no native Wolbachia infection [223]. That Ae. aegypti has no native Wolbachia infection affords the opportunity to transform populations with a Wolbachia strain of choice. When introduced to a new host, Wolbachia can provide pathogen protection that was not apparent in the native host, or is stronger than was found in the native host. For example, the wAlbB strain blocks DENV proliferation significantly more in the non-native hosts Ae. polynesiensis and Ae. aegypti, as compared to the native host Ae. albopictus. [11,214]. The wPip strain from Cx. pipiens, when transferred to Ae. aegypti, protects against DENV infection, though at a significant cost to fitness [224]. While introduction of a “foreign” Wolbachia strain often results in up-regulated immunity in the new host, immune priming seems to not be the root cause of pathogen-blocking in native hosts [75,209]. Wolbachia titers are frequently higher in introduced hosts, resulting in more cellular stress [209,215,225], which likely explains the appearance of the blocking phenotype. Indeed, wAlbB titers can be as much as 80 times higher in the introduced host Ae. aegypti, as compared to the native host Ae. albopictus [210]. The same holds true for transfers of Wolbachia between Drosophila species. Wolbachia titer correlates with the ability to protect against pathogens [87], and titers often change significantly upon introduction to a new host [215,226].

To further confound the comparison of native and non-native host-Wolbachia associations, non-native Wolbachia infections have been studied both as transient somatic infections, and as stable maternally transmitted infections. There is evidence of transient somatic infections that have enhanced pathogen replication. For example, wAlbB transfected into Culex tarsalis resulted in enhanced WNV infection [227]. However, there are limited data comparing these Wolbachia infection models. A separate study followed up on that finding, and compared stable and transient wAlbB infections in Ae. aegypti, challenged with WNV [228]. Here, the stable and transient infection models agreed with each other, both inhibiting WNV infection, which is contrary to the wAlbB-Cx. tarsalis-WNV finding of WNV enhancement. It is clear that there are many interacting genetic and environmental factors that affect the expression of pathogen-blocking.

## 7. Conclusions

There is considerable overlap in the obligate intracellular lives of *Wolbachia* and viruses. Intracellular invasion and trafficking, relying on the host cell for molecular building blocks and energy, and the necessity to evade host defenses have all resulted in convergent infection phenotypes, including reliance on host membranes and the induction of cellular stress. Our current understanding of the pathogen-blocking phenotype supports the idea that *Wolbachia* infection has modified the intracellular environment of the host such that it is refractory to viral replication. Indeed, there are many steps in the viral life cycle that require host processes, structures, or molecules that would already be perturbed by *Wolbachia*’s presence upon viral entry.

The previously proposed pathogen-blocking mechanisms can be united under the umbrella of cellular stress. Even seemingly unrelated mechanisms (e.g., Dnmt2 vs. cholesterol) may well be related. For example, altered membrane structure, cholesterol availability, or the formation of stress granules may mediate changes in the localization or activity of Dnmt2 or other host factors. Many of the immune and signaling pathways are interconnected and reliant upon specific ROS or metabolite conditions. Relative levels of cellular stress may also help to explain discrepancies in the manifestation of pathogen-blocking across host-*Wolbachia* associations. Certainly, native and non-native host-*Wolbachia* associations result in different infection phenotypes including differences in immune pathway regulation and *Wolbachia* titers.

Moving forward, it will be important to consider both the evolutionary history of specific *Wolbachia*-host associations, and the potential for cumulative or synergistic roadblocks affecting viral replication. Controlled comparisons of native and non-native *Wolbachia*-host associations will be useful for dissecting the link between cellular stress and pathogen-blocking. Additionally, native and non-native comparisons will generate a better understanding of how *Wolbachia* transfections might affect the selective pressures of the symbiosis, and of the pathogen-blocking phenotype. Attention to the specific cellular and molecular requirements of viral pathogens, as well as specific steps in the viral life cycle will help narrow down the times and places in which blocking occurs. Indeed, something like perturbed cholesterol may affect several steps in a virus’ lifecycle. Examining the intracellular localization of key players such as Dnmt2, viral replication complexes, and stress granules will further contribute to developing a deeper understanding of how *Wolbachia* infection results in pathogen-blocking. A thorough understanding of the mechanisms of pathogen-blocking across different *Wolbachia*-host associations will benefit the development and maintenance of *Wolbachia*-based vector control programs.

## References

[1] Pialoux G., Gaüzère B.-A., Jauréguiberry S., Strobel M. (2007). Chikungunya, an epidemic arbovirosis. Lancet Infect. Dis..

[2] Pybus O.G., Rambaut A. (2009). Evolutionary analysis of the dynamics of viral infectious disease. Nat. Rev. Genet..

[3] Moya A., Holmes E.C., González-Candelas F. (2004). The population genetics and evolutionary epidemiology of RNA viruses. Nat. Rev. Microbiol..

[4] Grenfell B.T., Pybus O.G., Gog J.R., Wood J.L., Daly J.M., Mumford J.A., Holmes E.C. (2004). Unifying the epidemiological and evolutionary dynamics of pathogens. Science.

[5] Lazear H.M., Diamond M.S. (2016). Zika virus: New clinical syndromes and its emergence in the western hemisphere. J. Virol..

[6] Makungu C., Stephen S., Kumburu S., Govella N.J., Dongus S., Hildon Z.J.-L., Killeen G.F., Jones C. (2017). Informing new or improved vector control tools for reducing the malaria burden in Tanzania: A qualitative exploration of perceptions of mosquitoes and methods for their control among the residents of dar es salaam. Malar. J..

[7] Jansen C.C., Beebe N.W. (2010). The dengue vector *Aedes aegypti*: What comes next. Microbes Infect..

[8] Zug R., Hammerstein P. (2012). Still a host of hosts for *Wolbachia*: Analysis of recent data suggests that 40% of terrestrial arthropod species are infected. PLoS ONE.

[9] Hoffmann A.A., Ross P.A., Rasic G. (2015). *Wolbachia* strains for disease control: Ecological and evolutionary considerations. Ecol. Evol..

[10] Werren J.H., Baldo L., Clark M.E. (2008). *Wolbachia*: Master manipulators of invertebrate biology. Nat. Rev. Microbiol..

[11] Bian G.W., Xu Y., Lu P., Xie Y., Xi Z.Y. (2010). The endosymbiotic bacterium *Wolbachia* induces resistance to dengue virus in *Aedes aegypti*. PLoS Pathog..

[12] Teixeira L., Ferreira Á., Ashburner M. (2008). The bacterial symbiont *Wolbachia* induces resistance to RNA viral infections in *Drosophila melanogaster*. PLoS Biol..

[13] Frentiu F.D., Zakir T., Walker T., Popovici J., Pyke A.T., van den Hurk A., McGraw E.A., O’Neill S.L. (2014). Limited dengue virus replication in field-collected *Aedes aegypti* mosquitoes infected with *Wolbachia*. PLoS Negl. Trop. Dis..

[14] Walker T., Johnson P.H., Moreira L.A., Iturbe-Ormaetxe I., Frentiu F.D., McMeniman C.J., Leong Y.S., Dong Y., Axford J., Kriesner P. (2011). The *w*Mel *Wolbachia* strain blocks dengue and invades caged *Aedes aegypti* populations. Nature.

[15] Carrington L.B., Tran B.C.N., Le N.T.H., Luong T.T.H., Nguyen T.T., Nguyen P.T., Nguyen C.V.V., Nguyen H.T.C., Vu T.T., Vo L.T. (2018). Field-and clinically derived estimates of *Wolbachia*-mediated blocking of dengue virus transmission potential in *Aedes aegypti* mosquitoes. Proc. Natl. Acad. Sci. USA.

[16] Turelli M., Hoffmann A.A. (1991). Rapid spread of an inherited incompatibility factor in California *Drosophila*. Nature.

[17] Turelli M., Hoffmann A.A. (1995). Cytoplasmic incompatibility in *Drosophila simulans*: Dynamics and parameter estimates from natural populations. Genetics.

[18] Hamm C.A., Begun D.J., Vo A., Smith C.C., Saelao P., Shaver A.O., Jaenike J., Turelli M. (2014). *Wolbachia* do not live by reproductive manipulation alone: Infection polymorphism in *Drosophila suzukii* and *D. subpulchrella*. Mol. Ecol..

[19] Stouthamer R., Breeuwer J.A.J., Luck R.F., Werren J.H. (1993). Molecular-identification of microorganisms associated with parthenogenesis. Nature.

[20] Stouthamer R., Luck R.F., Hamilton W.D. (1990). Antibiotics cause parthenogenetic *Trichogramma* (*Hymenoptera*, *trichogrammatidae*) to revert to sex. Proc. Natl. Acad. Sci. USA.

[21] Rigaud T., Juchault P. (1993). Conflict between feminizing sex ratio distorters and an autosomal masculinizing gene in the terrestrial isopod *Armadillidium vulgare* LATR. Genetics.

[22] Hurst G.D., Jiggins F.M., von der Schulenburg J.H.G., Bertrand D., West S.A., Goriacheva I.I., Zakharov I.A., Werren J.H., Stouthamer R., Majerus M.E. (1999). Male–killing *Wolbachia* in two species of insect. Proc. R. Soc. B Biol. Sci..

[23] Yen J.H., Barr A.R. (1971). New hypothesis of the cause of cytoplasmic incompatibility in *Culex pipiens*. Nature.

[24] Landmann F., Orsi G.A., Loppin B., Sullivan W. (2009). *Wolbachia*-mediated cytoplasmic incompatibility is associated with impaired histone deposition in the male pronucleus. PLoS Pathog..

[25] Lassy C.W., Karr T.L. (1996). Cytological analysis of fertilization and early embryonic development in incompatible crosses of *Drosophila simulans*. Mech. Dev..

[26] Serbus L.R., Casper-Lindley C., Landmann F., Sullivan W. (2008). The genetics and cell biology of *Wolbachia*-host interactions. Annu. Rev. Genet..

[27] Tram U., Sullivan W. (2002). Role of delayed nuclear envelope breakdown and mitosis in *Wolbachia*-induced cytoplasmic incompatibility. Science.

[28] LePage D.P., Metcalf J.A., Bordenstein S.R., On J., Perlmutter J.I., Shropshire J.D., Layton E.M., Funkhouser-Jones L.J., Beckmann J.F., Bordenstein S.R. (2017). Prophage WO genes recapitulate and enhance *Wolbachia*-induced cytoplasmic incompatibility. Nature.

[29] Beckmann J.F., Ronau J.A., Hochstrasser M. (2017). A *Wolbachia* deubiquitylating enzyme induces cytoplasmic incompatibility. Nat. Microbiol..

[30] Laven H. (1967). Eradication of *Culex pipiens fatigans* through cytoplasmic incompatibility. Nature.

[31] Min K.-T., Benzer S. (1997). *Wolbachia*, normally a symbiont of *Drosophila*, can be virulent, causing degeneration and early death. Proc. Natl. Acad. Sci. USA.

[32] McMeniman C.J., Lane R.V., Cass B.N., Fong A.W., Sidhu M., Wang Y.-F., O’neill S.L. (2009). Stable introduction of a life-shortening *Wolbachia* infection into the mosquito *Aedes aegypti*. Science.

[33] Yeap H.L., Axford J.K., Popovici J., Endersby N.M., Iturbe-Ormaetxe I., Ritchie S.A., Hoffmann A.A. (2014). Assessing quality of life-shortening *Wolbachia*-infected *Aedes aegypti* mosquitoes in the field based on capture rates and morphometric assessments. Parasites Vectors.

[34] Hoffmann A.A., Montgomery B.L., Popovici J., Iturbe-Ormaetxe I., Johnson P.H., Muzzi F., Greenfield M., Durkan M., Leong Y.S., Dong Y. (2011). Successful establishment of *Wolbachia* in *Aedes* populations to suppress dengue transmission. Nature.

[35] Hoffmann A.A., Turelli M., Harshman L.G. (1990). Factors affecting the distribution of cytoplasmic incompatibility in *Drosophila simulans*. Genetics.

[36] Hoffmann A.A., Iturbe-Ormaetxe I., Callahan A.G., Phillips B.L., Billington K., Axford J.K., Montgomery B., Turley A.P., O’Neill S.L. (2014). Stability of the *w*Mel *Wolbachia* infection following invasion into *Aedes aegypti* populations. PLoS Negl. Trop. Dis..

[37] Yixin H.Y., Carrasco A.M., Frentiu F.D., Chenoweth S.F., Beebe N.W., van den Hurk A.F., Simmons C.P., O’Neill S.L., McGraw E.A. (2015). *Wolbachia* reduces the transmission potential of dengue-infected *Aedes aegypti*. PLoS Negl. Trop. Dis..

[38] Lindsey A.R.I., Rice D.W., Bordenstein S.R., Brooks A.W., Bordenstein S.R., Newton I.L.G. (2018). Evolutionary genetics of cytoplasmic incompatibility genes *cifA* and *cifB* in prophage WO of *Wolbachia*. Genome Biol. Evol..

[39] Dobson S.L., Bourtzis K., Braig H.R., Jones B.F., Zhou W., Rousset F., O’Neill S.L. (1999). *Wolbachia* infections are distributed throughout insect somatic and germ line tissues. Insect Biochem. Mol. Biol..

[40] Pietri J.E., DeBruhl H., Sullivan W. (2016). The rich somatic life of *Wolbachia*. Microbiol. Open.

[41] Xi Z., Gavotte L., Xie Y., Dobson S.L. (2008). Genome-wide analysis of the interaction between the endosymbiotic bacterium *Wolbachia* and its *Drosophila* host. BMC Genom..

[42] Zheng Y., Wang J.L., Liu C., Wang C.P., Walker T., Wang Y.F. (2011). Differentially expressed profiles in the larval testes of *Wolbachia* infected and uninfected *Drosophila*. BMC Genom..

[43] Kremer N., Charif D., Henri H., Gavory F., Wincker P., Mavingui P., Vavre F. (2012). Influence of *Wolbachia* on host gene expression in an obligatory symbiosis. BMC Microbiol..

[44] Hughes G.L., Ren X.X., Ramirez J.L., Sakamoto J.M., Bailey J.A., Jedlicka A.E., Rasgon J.L. (2011). *Wolbachia* infections in *Anopheles gambiae* cells: Transcriptomic characterization of a novel host-symbiont interaction. PLoS Pathog..

[45] Kremer N., Voronin D., Charif D., Mavingui P., Mollereau B., Vavre F. (2009). *Wolbachia* interferes with ferritin expression and iron metabolism in insects. PLoS Pathog..

[46] Molloy J.C., Sommer U., Viant M.R., Sinkins S.P. (2016). *Wolbachia* modulates lipid metabolism in *Aedes albopictus* mosquito cells. Appl. Environ. Microbiol..

[47] Stouthamer R., Luck R. (1993). Influence of microbe-associated parthenogenesis on the fecundity of *Trichogramma deion* and *T. Pretiosum*. Entomol. Exp. Appl..

[48] Dean M.D. (2006). A *Wolbachia*-associated fitness benefit depends on genetic background in *Drosophila simulans*. Proc. R. Soc. B Biol. Sci..

[49] Vasquez C.J., Stouthamer R., Jeong G., Morse J.G. (2011). Discovery of a ci-inducing *Wolbachia* and its associated fitness costs in the biological control agent *Aphytis melinus* debach (*Hymenoptera*: *Aphelinidae*). Biol. Control.

[50] Moreau J., Bertin A., Caubet Y., Rigaud T. (2001). Sexual selection in an isopod with *Wolbachia*-induced sex reversal: Males prefer real females. J. Evol. Biol..

[51] Jaenike J., Dyer K.A., Cornish C., Minhas M.S. (2006). Asymmetrical reinforcement and *Wolbachia* infection in *Drosophila*. PLoS Biol..

[52] Bordenstein S.R., O’hara F.P., Werren J.H. (2001). *Wolbachia*-induced incompatibility precedes other hybrid incompatibilities in *Nasonia*. Nature.

[53] Cho K.-O., Kim G.-W., Lee O.-K. (2011). *Wolbachia* bacteria reside in host golgi-related vesicles whose position is regulated by polarity proteins. PLoS ONE.

[54] White P.M., Serbus L.R., Debec A., Codina A., Bray W., Guichet A., Lokey R.S., Sullivan W. (2017). Reliance of *Wolbachia* on high rates of host proteolysis revealed by a genome-wide RNAi screen of *Drosophila* cells. Genetics.

[55] Geoghegan V., Stainton K., Rainey S.M., Ant T.H., Dowle A.A., Larson T., Hester S., Charles P.D., Thomas B., Sinkins S.P. (2017). Perturbed cholesterol and vesicular trafficking associated with dengue blocking in *Wolbachia*-infected *Aedes aegypti* cells. Nat. Commun..

[56] Caragata E.P., Rancès E., Hedges L.M., Gofton A.W., Johnson K.N., O’Neill S.L., McGraw E.A. (2013). Dietary cholesterol modulates pathogen blocking by *Wolbachia*. PLoS Pathog..

[57] Ferree P.M., Frydman H.M., Li J.M., Cao J., Wieschaus E., Sullivan W. (2005). *Wolbachia* utilizes host microtubules and dynein for anterior localization in the *Drosophila* oocyte. PLoS Pathog..

[58] Sheehan K.B., Martin M., Lesser C.F., Isberg R.R., Newton I.L. (2016). Identification and characterization of a candidate *Wolbachia pipientis* type IV effector that interacts with the actin cytoskeleton. MBio.

[59] Newton I.L., Savytskyy O., Sheehan K.B. (2015). *Wolbachia* utilize host actin for efficient maternal transmission in *Drosophila melanogaster*. PLoS Pathog..

[60] Bhattacharya T., Newton I.L. (2017). Mi casa es su casa: How an intracellular symbiont manipulates host biology. Environ. Microbiol..

[61] Rice D.W., Sheehan K.B., Newton I.L. (2017). Large-scale identification of *Wolbachia pipientis* effectors. Genome Biol. Evol..

[62] Ote M., Ueyama M., Yamamoto D. (2016). *Wolbachia* protein tomo targets nanos mRNA and restores germ stem cells in *Drosophila* sex-lethal mutants. Curr. Biol..

[63] Brennan L.J., Keddie B.A., Braig H.R., Harris H.L. (2008). The endosymbiont *Wolbachia pipientis* induces the expression of host antioxidant proteins in an *Aedes albopictus* cell line. PLoS ONE.

[64] Mayoral J.G., Etebari K., Hussain M., Khromykh A.A., Asgari S. (2014). *Wolbachia* infection modifies the profile, shuttling and structure of microRNAs in a mosquito cell line. PLoS ONE.

[65] Osei-Amo S., Hussain M., O’Neill S.L., Asgari S. (2012). *Wolbachia*-induced aae-mir-12 miRNA negatively regulates the expression of MCT1 and MCM6 genes in *Wolbachia*-infected mosquito cell line. PLoS ONE.

[66] Hussain M., Frentiu F.D., Moreira L.A., O’Neill S.L., Asgari S. (2011). *Wolbachia* uses host microRNAs to manipulate host gene expression and facilitate colonization of the dengue vector *Aedes aegypti*. Proc. Natl. Acad. Sci. USA.

[67] Pinto S.B., Mariconti M., Bazzocchi C., Bandi C., Sinkins S.P. (2012). *Wolbachia* surface protein induces innate immune responses in mosquito cells. BMC Microbiol..

[68] Ye Y.X.H., Woolfit M., Huttley G.A., Rances E., Caragata E.P., Popovici J., O’Neill S.L., McGraw E.A. (2013). Infection with a virulent strain of *Wolbachia* disrupts genome wide-patterns of cytosine methylation in the mosquito *Aedes aegypti*. PLoS ONE.

[69] LePage D.P., Jernigan K.K., Bordenstein S.R. (2014). The relative importance of DNA methylation and DNMT2-mediated epigenetic regulation on *Wolbachia* densities and cytoplasmic incompatibility. PeerJ.

[70] Negri H., Franchini A., Gonella E., Daffonchio D., Mazzoglio P.J., Mandrioli M., Alma A. (2009). Unravelling the *Wolbachia* evolutionary role: The reprogramming of the host genomic imprinting. Proc. R. Soc. B Biol. Sci..

[71] Wu M., Sun L.V., Vamathevan J., Riegler M., Deboy R., Brownlie J.C., McGraw E.A., Martin W., Esser C., Ahmadinejad N. (2004). Phylogenomics of the reproductive parasite *Wolbachia* pipientis *w*Mel: A streamlined genome overrun by mobile genetic elements. PLoS Biol..

[72] Caragata E.P., Rancès E., O’Neill S.L., McGraw E.A. (2014). Competition for amino acids between *Wolbachia* and the mosquito host, *Aedes aegypti*. Microb. Ecol..

[73] Brownlie J.C., Cass B.N., Riegler M., Witsenburg J.J., Iturbe-Ormaetxe I., McGraw E.A., O’Neill S.L. (2009). Evidence for metabolic provisioning by a common invertebrate endosymbiont, *Wolbachia pipientis*, during periods of nutritional stress. PLoS Pathog..

[74] Gerth M., Bleidorn C. (2016). Comparative genomics provides a timeframe for *Wolbachia* evolution and exposes a recent biotin synthesis operon transfer. Nat. Microbiol..

[75] Rancès E., Ye Y.H., Woolfit M., McGraw E.A., O’Neill S.L. (2012). The relative importance of innate immune priming in *Wolbachia*-mediated dengue interference. PLoS Pathog..

[76] Bhattacharya T., Newton I.L.G., Hardy R.W. (2017). *Wolbachia* elevates host methyltransferase expression to block an RNA virus early during infection. PLOS Pathog..

[77] Glaser R.L., Meola M.A. (2010). The native *Wolbachia* endosymbionts of *Drosophila melanogaster* and *Culex quinquefasciatus* increase host resistance to west nile virus infection. PLoS ONE.

[78] Rainey S.M., Martinez J., McFarlane M., Juneja P., Sarkies P., Lulla A., Schnettler E., Varjak M., Merits A., Miska E.A. (2016). *Wolbachia* blocks viral genome replication early in infection without a transcriptional response by the endosymbiont or host small RNA pathways. PLoS Pathog..

[79] Van den Hurk A.F., Hall-Mendelin S., Pyke A.T., Frentiu F.D., McElroy K., Day A., Higgs S., O’Neill S.L. (2012). Impact of *Wolbachia* on infection with chikungunya and yellow fever viruses in the mosquito vector *Aedes aegypti*. PLoS Negl. Trop. Dis..

[80] Blagrove M.S., Arias-Goeta C., Di Genua C., Failloux A.-B., Sinkins S.P. (2013). A *Wolbachia w*Mel transinfection in *Aedes albopictus* is not detrimental to host fitness and inhibits chikungunya virus. PLoS Negl. Trop. Dis..

[81] Moreira L.A., Iturbe-Ormaetxe I., Jeffery J.A., Lu G., Pyke A.T., Hedges L.M., Rocha B.C., Hall-Mendelin S., Day A., Riegler M. (2009). A *Wolbachia* symbiont in *Aedes aegypti* limits infection with dengue, chikungunya, and *Plasmodium*. Cell.

[82] Aliota M.T., Peinado S.A., Velez I.D., Osorio J.E. (2016). The *w*Mel strain of *Wolbachia* reduces transmission of zika virus by *Aedes aegypti*. Sci. Rep..

[83] Dutra H.L.C., Rocha M.N., Dias F.B.S., Mansur S.B., Caragata E.P., Moreira L.A. (2016). *Wolbachia* blocks currently circulating zika virus isolates in brazilian *Aedes aegypti* mosquitoes. Cell Host Microbe.

[84] Graham R.I., Grzywacz D., Mushobozi W.L., Wilson K. (2012). *Wolbachia* in a major African crop pest increases susceptibility to viral disease rather than protects. Ecol. Lett..

[85] Osborne S.E., Leong Y.S., O’Neill S.L., Johnson K.N. (2009). Variation in antiviral protection mediated by different *Wolbachia* strains in *Drosophila simulans*. PLoS Pathog..

[86] Chrostek E., Marialva M.S.P., Esteves S.S., Weinert L.A., Martinez J., Jiggins F.M., Teixeira L. (2013). *Wolbachia* variants induce differential protection to viruses in *Drosophila melanogaster*: A phenotypic and phylogenomic analysis. PLoS Genet..

[87] Martinez J., Longdon B., Bauer S., Chan Y.-S., Miller W.J., Bourtzis K., Teixeira L., Jiggins F.M. (2014). Symbionts commonly provide broad spectrum resistance to viruses in insects: A comparative analysis of *Wolbachia* strains. PLoS Pathog..

[88] Schultz M.J., Isern S., Michael S.F., Corley R.B., Connor J., Frydman H.M. (2017). Variable inhibition of zika virus replication by different *Wolbachia* strains in mosquito cell cultures. J. Virol..

[89] Mousson L., Zouache K., Arias-Goeta C., Raquin V., Mavingui P., Failloux A.-B. (2012). The native *Wolbachia* symbionts limit transmission of dengue virus in *Aedes albopictus*. PLoS Negl. Trop. Dis..

[90] Ant T.H., Herd C.S., Geoghegan V., Hoffmann A.A., Sinkins S.P. (2018). The *Wolbachia* strain *w*Au provides highly efficient virus transmission blocking in *Aedes aegypti*. PLoS Pathog..

[91] Schnettler E., Sreenu V.B., Mottram T., McFarlane M. (2016). *Wolbachia* restricts insect-specific flavivirus infection in *Aedes aegypti* cells. J. Gen. Virol..

[92] Harrison S.C. (2015). Viral membrane fusion. Virology.

[93] Chandran K., Farsetta D.L., Nibert M.L. (2002). Strategy for nonenveloped virus entry: A hydrophobic conformer of the reovirus membrane penetration protein μ1 mediates membrane disruption. J. Virol..

[94] Danthi P., Kobayashi T., Holm G.H., Hansberger M.W., Abel T.W., Dermody T.S. (2008). Reovirus apoptosis and virulence are regulated by host cell membrane penetration efficiency. J. Virol..

[95] Lu Y.E., Cassese T., Kielian M. (1999). The cholesterol requirement for sindbis virus entry and exit and characterization of a spike protein region involved in cholesterol dependence. J. Virol..

[96] Kielian M.C., Helenius A. (1984). Role of cholesterol in fusion of semliki forest virus with membranes. J. Virol..

[97] Carro A.C., Damonte E.B. (2013). Requirement of cholesterol in the viral envelope for dengue virus infection. Virus Res..

[98] Belov G.A. (2014). Modulation of lipid synthesis and trafficking pathways by picornaviruses. Curr. Opin. Virol..

[99] Issac T.H.K., Tan E.L., Chu J.J.H. (2014). Proteomic profiling of chikungunya virus-infected human muscle cells: Reveal the role of cytoskeleton network in chikv replication. J. Proteom..

[100] Radtke K., Döhner K., Sodeik B. (2006). Viral interactions with the cytoskeleton: A hitchhiker’s guide to the cell. Cell Microbiol..

[101] Lai C.-K., Jeng K.-S., Machida K., Lai M.M. (2008). Association of hepatitis c virus replication complexes with microtubules and actin filaments is dependent on the interaction of NS3 and NS5A. J. Virol..

[102] Bernard E., Solignat M., Gay B., Chazal N., Higgs S., Devaux C., Briant L. (2010). Endocytosis of chikungunya virus into mammalian cells: Role of clathrin and early endosomal compartments. PLoS ONE.

[103] Welsch S., Miller S., Romero-Brey I., Merz A., Bleck C.K., Walther P., Fuller S.D., Antony C., Krijnse-Locker J., Bartenschlager R. (2009). Composition and three-dimensional architecture of the dengue virus replication and assembly sites. Cell Host Microbe.

[104] Gillespie L.K., Hoenen A., Morgan G., Mackenzie J.M. (2010). The endoplasmic reticulum provides the membrane platform for biogenesis of the flavivirus replication complex. J. Virol..

[105] Kujala P., Ikäheimonen A., Ehsani N., Vihinen H., Auvinen P., Kääriäinen L. (2001). Biogenesis of the semliki forest virus RNA replication complex. J. Virol..

[106] Salonen A., Vasiljeva L., Merits A., Magden J., Jokitalo E., Kääriäinen L. (2003). Properly folded nonstructural polyprotein directs the semliki forest virus replication complex to the endosomal compartment. J. Virol..

[107] Taylor M.P., Kirkegaard K. (2008). Potential subversion of autophagosomal pathway by picornaviruses. Autophagy.

[108] Kopek B.G., Perkins G., Miller D.J., Ellisman M.H., Ahlquist P. (2007). Three-dimensional analysis of a viral RNA replication complex reveals a virus-induced mini-organelle. PLoS Biol..

[109] Van Wynsberghe P.M., Ahlquist P. (2009). 5′ cis elements direct nodavirus RNA1 recruitment to mitochondrial sites of replication complex formation. J. Virol..

[110] Bost A.G., Venable D., Liu L., Heinz B.A. (2003). Cytoskeletal requirements for hepatitis c virus (HCV) RNA synthesis in the HCV replicon cell culture system. J. Virol..

[111] Barton D.J., Sawicki S.G., Sawicki D.L. (1991). Solubilization and immunoprecipitation of alphavirus replication complexes. J. Virol..

[112] Ng C.G., Coppens I., Govindarajan D., Pisciotta J., Shulaev V., Griffin D.E. (2008). Effect of host cell lipid metabolism on alphavirus replication, virion morphogenesis, and infectivity. Proc. Natl. Acad. Sci. USA.

[113] Poh M.K., Shui G., Xie X., Shi P.-Y., Wenk M.R., Gu F. (2012). U18666a, an intra-cellular cholesterol transport inhibitor, inhibits dengue virus entry and replication. Antivir. Res..

[114] Rothwell C., LeBreton A., Ng C.Y., Lim J.Y., Liu W., Vasudevan S., Labow M., Gu F., Gaither L.A. (2009). Cholesterol biosynthesis modulation regulates dengue viral replication. Virology.

[115] Heaton N.S., Perera R., Berger K.L., Khadka S., LaCount D.J., Kuhn R.J., Randall G. (2010). Dengue virus nonstructural protein 3 redistributes fatty acid synthase to sites of viral replication and increases cellular fatty acid synthesis. Proc. Natl. Acad. Sci. USA.

[116] Mackenzie J.M., Khromykh A.A., Parton R.G. (2007). Cholesterol manipulation by west nile virus perturbs the cellular immune response. Cell Host Microbe.

[117] Roosendaal J., Westaway E.G., Khromykh A., Mackenzie J.M. (2006). Regulated cleavages at the west nile virus NS4A-2K-NS4B junctions play a major role in rearranging cytoplasmic membranes and golgi trafficking of the NS4A protein. J. Virol..

[118] Ahlquist P., Noueiry A.O., Lee W.-M., Kushner D.B., Dye B.T. (2003). Host factors in positive-strand RNA virus genome replication. J. Virol..

[119] Ahlquist P. (2006). Parallels among positive-strand RNA viruses, reverse-transcribing viruses and double-stranded RNA viruses. Nat. Rev. Microbiol..

[120] Firth A.E., Brierley I. (2012). Non-canonical translation in RNA viruses. J. Gen. Virol..

[121] Gale M., Tan S.-L., Katze M.G. (2000). Translational control of viral gene expression in eukaryotes. Microbiol. Mol. Biol. Rev..

[122] Beckham C.J., Parker R. (2008). P bodies, stress granules, and viral life cycles. Cell Host Microbe.

[123] McInerney G.M., Kedersha N.L., Kaufman R.J., Anderson P., Liljeström P. (2005). Importance of EIF2A phosphorylation and stress granule assembly in alphavirus translation regulation. Mol. Biol. Cell.

[124] Panas M.D., Varjak M., Lulla A., Eng K.E., Merits A., Hedestam G.B.K., McInerney G.M. (2012). Sequestration of G3BP coupled with efficient translation inhibits stress granules in semliki forest virus infection. Mol. Biol. Cell.

[125] Cristea I.M., Rozjabek H., Molloy K.R., Karki S., White L.L., Rice C.M., Rout M.P., Chait B.T., MacDonald M.R. (2010). Host factors associated with the sindbis virus RNA-dependent RNA polymerase: Role for g3bp1 and g3bp2 in virus replication. J. Virol..

[126] Fros J.J., Domeradzka N.E., Baggen J., Geertsema C., Flipse J., Vlak J.M., Pijlman G.P. (2012). Chikungunya virus NSP3 blocks stress granule assembly by recruitment of G3BP into cytoplasmic foci. J. Virol..

[127] Pager C.T., Schütz S., Abraham T.M., Luo G., Sarnow P. (2013). Modulation of hepatitis C virus RNA abundance and virus release by dispersion of processing bodies and enrichment of stress granules. Virology.

[128] Rathore A.P., Ng M.-L., Vasudevan S.G. (2013). Differential unfolded protein response during chikungunya and sindbis virus infection: Chikv NSP4 suppresses EIF2A phosphorylation. Virol. J..

[129] Garcia-Moreno M., Sanz M.A., Pelletier J., Carrasco L. (2013). Requirements for EIF4A and EIF2 during translation of sindbis virus subgenomic mRNA in vertebrate and invertebrate host cells. Cell. Microbiol..

[130] Berlanga J.J., Ventoso I., Harding H.P., Deng J., Ron D., Sonenberg N., Carrasco L., de Haro C. (2006). Antiviral effect of the mammalian translation initiation factor 2α kinase GCN2 against RNA viruses. EMBO J..

[131] Pan X., Zhou G., Wu J., Bian G., Lu P., Raikhel A.S., Xi Z. (2012). *Wolbachia* induces reactive oxygen species (ROS)-dependent activation of the toll pathway to control dengue virus in the mosquito *Aedes aegypti*. Proc. Natl. Acad. Sci. USA.

[132] Junker-Niepmann M., Bartenschlager R., Schaller H. (1990). A short cis-acting sequence is required for hepatitis b virus pregenome encapsidation and sufficient for packaging of foreign RNA. EMBO J..

[133] Firth A.E., Atasheva S., Frolova E.I., Frolov I. (2011). Conservation of a packaging signal and the viral genome RNA packaging mechanism in alphavirus evolution. J. Virol..

[134] Yoshii K., Goto A., Kawakami K., Kariwa H., Takashima I. (2008). Construction and application of chimeric virus-like particles of tick-borne encephalitis virus and mosquito-borne japanese encephalitis virus. J. Gen. Virol..

[135] Gokhale N.S., McIntyre A.B., McFadden M.J., Roder A.E., Kennedy E.M., Gandara J.A., Hopcraft S.E., Quicke K.M., Vazquez C., Willer J. (2016). N6-methyladenosine in flaviviridae viral RNA genomes regulates infection. Cell Host Microbe.

[136] Lichinchi G., Zhao B.S., Wu Y., Lu Z., Qin Y., He C., Rana T.M. (2016). Dynamics of human and viral RNA methylation during zika virus infection. Cell Host Microbe.

[137] Bhattacharya B., Roy P. (2010). Role of lipids on entry and exit of bluetongue virus, a complex non-enveloped virus. Viruses.

[138] Martínez-Gutierrez M., Castellanos J.E., Gallego-Gómez J.C. (2011). Statins reduce dengue virus production via decreased virion assembly. Intervirology.

[139] Suthar M.S., Diamond M.S., Gale M. (2013). West nile virus infection and immunity. Nat. Rev. Microbiol..

[140] Diamond M.S., Shrestha B., Mehlhop E., Sitati E., Engle M. (2003). Innate and adaptive immune responses determine protection against disseminated infection by west nile encephalitis virus. Viral Immunol..

[141] Harley D., Sleigh A., Ritchie S. (2001). Ross river virus transmission, infection, and disease: A cross-disciplinary review. Clin. Microbiol. Rev..

[142] Ryman K.D., Klimstra W.B. (2008). Host responses to alphavirus infection. Immunol. Rev..

[143] Lemaitre B., Hoffmann J. (2007). The host defense of *Drosophila melanogaster*. Annual Review of Immunology.

[144] Tassetto M., Kunitomi M., Andino R. (2017). Circulating immune cells mediate a systemic RNAi-based adaptive antiviral response in *Drosophila*. Cell.

[145] Blair C.D., Olson K.E. (2015). The role of RNA interference (RNAi) in arbovirus-vector interactions. Viruses.

[146] Han Y.-H., Luo Y.-J., Wu Q., Jovel J., Wang X.-H., Aliyari R., Han C., Li W.-X., Ding S.-W. (2011). RNA-based immunity terminates viral infection in adult *Drosophila* in the absence of viral suppression of RNA interference: Characterization of viral small interfering RNA populations in wild-type and mutant flies. J. Virol..

[147] Junjhon J., Pennington J.G., Edwards T.J., Perera R., Lanman J., Kuhn R.J. (2014). Ultrastructural characterization and three-dimensional architecture of replication sites in dengue virus-infected mosquito cells. J. Virol..

[148] Brackney D.E., Beane J.E., Ebel G.D. (2009). Rnai targeting of west nile virus in mosquito midguts promotes virus diversification. PLoS Pathog..

[149] Siu R.W., Fragkoudis R., Simmonds P., Donald C.L., Chase-Topping M.E., Barry G., Attarzadeh-Yazdi G., Rodriguez-Andres J., Nash A.A., Merits A. (2011). Antiviral RNA interference responses induced by semliki forest virus infection of mosquito cells: Characterization, origin, and frequency-dependent functions of virus-derived small interfering RNAs. J. Virol..

[150] Michel T., Reichhart J.-M., Hoffmann J.A., Royet J. (2001). *Drosophila* toll is activated by gram-positive bacteria through a circulating peptidoglycan recognition protein. Nature.

[151] Zambon R.A., Nandakumar M., Vakharia V.N., Wu L.P. (2005). The toll pathway is important for an antiviral response in *Drosophila*. Proc. Natl. Acad. Sci. USA.

[152] Xi Z., Ramirez J.L., Dimopoulos G. (2008). The *Aedes aegypti* toll pathway controls dengue virus infection. PLoS Pathog..

[153] Kaneko T., Silverman N. (2005). Bacterial recognition and signalling by the *Drosophila* IMD pathway. Cell. Microbiol..

[154] Costa A., Jan E., Sarnow P., Schneider D. (2009). The IMD pathway is involved in antiviral immune responses in *Drosophila*. PLoS ONE.

[155] Kingsolver M.B., Huang Z., Hardy R.W. (2013). Insect antiviral innate immunity: Pathways, effectors, and connections. J. Mol. Biol..

[156] Xu J., Cherry S. (2014). Viruses and antiviral immunity in *Drosophila*. Dev. Comp. Immunol..

[157] Avadhanula V., Weasner B.P., Hardy G.G., Kumar J.P., Hardy R.W. (2009). A novel system for the launch of alphavirus RNA synthesis reveals a role for the imd pathway in arthropod antiviral response. PLoS Pathog..

[158] Fragkoudis R., Chi Y., Siu R., Barry G., Attarzadeh-Yazdi G., Merits A., Nash A., Fazakerley J., Kohl A. (2008). Semliki forest virus strongly reduces mosquito host defense signaling. Insect Mol. Biol..

[159] Huang Z., Kingsolver M.B., Avadhanula V., Hardy R.W. (2013). An antiviral role for antimicrobial peptides during the arthropod response to alphavirus replication. J. Virol..

[160] Kemp C., Mueller S., Goto A., Barbier V., Paro S., Bonnay F., Dostert C., Troxler L., Hetru C., Meignin C. (2013). Broad RNA interference–Mediated antiviral immunity and virus-specific inducible responses in *Drosophila*. J. Immunol..

[161] Dostert C., Jouanguy E., Irving P., Troxler L., Galiana-Arnoux D., Hetru C., Hoffmann J.A., Imler J.-L. (2005). The JAK-STAT signaling pathway is required but not sufficient for the antiviral response of *Drosophila*. Nat. Immunol..

[162] Deddouche S., Matt N., Budd A., Mueller S., Kemp C., Galiana-Arnoux D., Dostert C., Antoniewski C., Hoffmann J.A., Imler J.-L. (2008). The dexd/h-box helicase dicer-2 mediates the induction of antiviral activity in *Drosophila*. Nat. Immunol..

[163] Yoneyama M., Kikuchi M., Natsukawa T., Shinobu N., Imaizumi T., Miyagishi M., Taira K., Akira S., Fujita T. (2004). The RNA helicase RIG-I has an essential function in double-stranded RNA-induced innate antiviral responses. Nat. Immunol..

[164] Buchon N., Broderick N.A., Poidevin M., Pradervand S., Lemaitre B. (2009). *Drosophila* intestinal response to bacterial infection: Activation of host defense and stem cell proliferation. Cell Host Microbe.

[165] Barillas-Mury C., Han Y.S., Seeley D., Kafatos F.C. (1999). *Anopheles gambiae* ag-stat, a new insect member of the stat family, is activated in response to bacterial infection. EMBO J..

[166] Souza-Neto J.A., Sim S., Dimopoulos G. (2009). An evolutionary conserved function of the JAK-STAT pathway in anti-dengue defense. Proc. Natl. Acad. Sci. USA.

[167] Paradkar P.N., Trinidad L., Voysey R., Duchemin J.-B., Walker P.J. (2012). Secreted vago restricts west nile virus infection in culex mosquito cells by activating the JAK-STAT pathway. Proc. Natl. Acad. Sci. USA.

[168] Arjona A., Ledizet M., Anthony K., Bonafé N., Modis Y., Town T., Fikrig E. (2007). West nile virus envelope protein inhibits dsRNA-induced innate immune responses. J. Immunol..

[169] Morrison J., Aguirre S., Fernandez-Sesma A. (2012). Innate immunity evasion by dengue virus. Viruses.

[170] Wong Z.S., Brownlie J.C., Johnson K.N. (2015). Oxidative stress correlates with *Wolbachia*-mediated antiviral protection in *Wolbachia*-*Drosophila* associations. Appl. Environ. Microbiol..

[171] Brennan L., Haukedal J., Earle J., Keddie B., Harris H. (2012). Disruption of redox homeostasis leads to oxidative DNA damage in spermatocytes of *Wolbachia*-infected *Drosophila simulans*. Insect Mol. Biol..

[172] Thannickal V.J., Fanburg B.L. (2000). Reactive oxygen species in cell signaling. Am. J. Physiol.-Lung Cell. Mol. Physiol..

[173] Müller J.M., Cahill M.A., Rupec R.A., Baeuerle P.A., Nordheim A. (1997). Antioxidants as well as oxidants activate c-fos via ras-dependent activation of extracellular-signal-regulated kinase 2 and ELK-1. FEBS J..

[174] Milligan S.A., Owens M.W., Grisham M.B. (1998). Differential regulation of extracellular signal-regulated kinase and nuclear factor-κb signal transduction pathways by hydrogen peroxide and tumor necrosis factor. Arch. Biochem. Biophys..

[175] Shaul Y.D., Seger R. (2007). The mek/erk cascade: From signaling specificity to diverse functions. Biochim. Biophys. Acta.

[176] Xu J., Hopkins K., Sabin L., Yasunaga A., Subramanian H., Lamborn I., Gordesky-Gold B., Cherry S. (2013). Erk signaling couples nutrient status to antiviral defense in the insect gut. Proc. Natl. Acad. Sci. USA.

[177] Sansone C.L., Cohen J., Yasunaga A., Xu J., Osborn G., Subramanian H., Gold B., Buchon N., Cherry S. (2015). Microbiota-dependent priming of antiviral intestinal immunity in *Drosophila*. Cell Host Microbe.

[178] Wong Z.S., Brownlie J.C., Johnson K.N. (2016). Impact of erk activation on fly survival and *Wolbachia*-mediated protection during virus infection. J. Gen. Virol..

[179] Werstuck G.H., Lentz S.R., Dayal S., Hossain G.S., Sood S.K., Shi Y.Y., Zhou J., Maeda N., Krisans S.K., Malinow M.R. (2001). Homocysteine-induced endoplasmic reticulum stress causes dysregulation of the cholesterol and triglyceride biosynthetic pathways. J. Clin. Investig..

[180] Lee J.-S., Mendez R., Heng H.H., Yang Z.-Q., Zhang K. (2012). Pharmacological ER stress promotes hepatic lipogenesis and lipid droplet formation. Am. J. Transl. Res..

[181] Lee C.-J., Lin H.-R., Liao C.-L., Lin Y.-L. (2008). Cholesterol effectively blocks entry of flavivirus. J. Virol..

[182] Sasao F., Igarashi A., Fukai K. (1980). Amino acid requirements for the growth of *Aedes albopictus* clone c6/36 cells and for the production of dengue and chikungunya viruses in the infected cells. Microbiol. Immunol..

[183] Harding H.P., Zhang Y., Zeng H., Novoa I., Lu P.D., Calfon M., Sadri N., Yun C., Popko B., Paules R. (2003). An integrated stress response regulates amino acid metabolism and resistance to oxidative stress. Mol. Cell.

[184] Durdevic Z., Hanna K., Gold B., Pollex T., Cherry S., Lyko F., Schaefer M. (2013). Efficient RNA virus control in *Drosophila* requires the RNA methyltransferase DNMT2. EMBO Rep..

[185] Durdevic Z., Mobin M.B., Hanna K., Lyko F., Schaefer M. (2013). The RNA methyltransferase DNMT2 is required for efficient dicer-2-dependent siRNA pathway activity in *Drosophila*. Cell Rep..

[186] Dubin D.T., Stollar V., Hsuchen C.-C., Timko K., Guild G.M. (1977). Sindbis virus messenger RNA: The 5′-termini and methylated residues of 26 and 42 s RNA. Virology.

[187] Zhang G., Hussain M., O’Neill S.L., Asgari S. (2013). *Wolbachia* uses a host microRNA to regulate transcripts of a methyltransferase, contributing to dengue virus inhibition in *Aedes aegypti*. Proc. Natl. Acad. Sci. USA.

[188] Gilbert W.V., Bell T.A., Schaening C. (2016). Messenger RNA modifications: Form, distribution, and function. Science.

[189] McIntyre W., Netzband R., Bonenfant G., Biegel J.M., Miller C., Fuchs G., Henderson E., Arra M., Canki M., Fabris D. (2018). Positive-sense RNA viruses reveal the complexity and dynamics of the cellular and viral epitranscriptomes during infection. Nucleic Acids Res..

[190] Goll M.G., Kirpekar F., Maggert K.A., Yoder J.A., Hsieh C.-L., Zhang X., Golic K.G., Jacobsen S.E., Bestor T.H. (2006). Methylation of trnaasp by the DNA methyltransferase homolog DNMT2. Science.

[191] Frolov I., Hardy R., Rice C.M. (2001). Cis-acting RNA elements at the 5′ end of sindbis virus genome RNA regulate minus-and plus-strand RNA synthesis. RNA.

[192] Khromykh A.A., Meka H., Guyatt K.J., Westaway E.G. (2001). Essential role of cyclization sequences in flavivirus RNA replication. J. Virol..

[193] Brinton M.A., Dispoto J.H. (1988). Sequence and secondary structure analysis of the 5′-terminal region of flavivirus genome RNA. Virology.

[194] Clyde K., Harris E. (2006). RNA secondary structure in the coding region of dengue virus type 2 directs translation start codon selection and is required for viral replication. J. Virol..

[195] Schaefer M., Pollex T., Hanna K., Tuorto F., Meusburger M., Helm M., Lyko F. (2010). RNA methylation by DNMT2 protects transfer RNAs against stress-induced cleavage. Genes Dev..

[196] Thiagarajan D., Dev R.R., Khosla S. (2011). The DNA methyltranferase DNMT2 participates in RNA processing during cellular stress. Epigenetics.

[197] Schaefer M., Lyko F. (2010). Solving the dnmt2 enigma. Chromosoma.

[198] Marhold J., Rothe N., Pauli A., Mund C., Kuehle K., Brueckner B., Lyko F. (2004). Conservation of DNA methylation in dipteran insects. Insect Mol. Biol..

[199] Hedges L.M., Yamada R., O’Neill S.L., Johnson K.N. (2012). The small interfering RNA pathway is not essential for *Wolbachia*-mediated antiviral protection in *Drosophila melanogaster*. Appl. Environ. Microbiol..

[200] Terradas G., Joubert D.A., McGraw E.A. (2017). The RNAi pathway plays a small part in *Wolbachia*-mediated blocking of dengue virus in mosquito cells. Sci. Rep..

[201] Lim D.-H., Oh C.-T., Lee L., Hong J.-S., Noh S.-H., Hwang S., Kim S., Han S.-J., Lee Y.S. (2011). The endogenous siRNA pathway in *Drosophila* impacts stress resistance and lifespan by regulating metabolic homeostasis. FEBS Lett..

[202] Vagin V.V., Sigova A., Li C., Seitz H., Gvozdev V., Zamore P.D. (2006). A distinct small RNA pathway silences selfish genetic elements in the germline. Science.

[203] Weber F., Wagner V., Rasmussen S.B., Hartmann R., Paludan S.R. (2006). Double-stranded RNA is produced by positive-strand RNA viruses and DNA viruses but not in detectable amounts by negative-strand RNA viruses. J. Virol..

[204] Kambris Z., Cook P.E., Phuc H.K., Sinkins S.P. (2009). Immune activation by life-shortening *Wolbachia* and reduced filarial competence in mosquitoes. Science.

[205] Wong Z.S., Hedges L.M., Brownlie J.C., Johnson K.N. (2011). *Wolbachia*-mediated antibacterial protection and immune gene regulation in *Drosophila*. PLoS ONE.

[206] Libert S., Chao Y., Chu X., Pletcher S.D. (2006). Trade-offs between longevity and pathogen resistance in *Drosophila melanogaster* are mediated by NFκB signaling. Aging Cell.

[207] Rancès E., Johnson T.K., Popovici J., Iturbe-Ormaetxe I., Zakir T., Warr C.G., O’Neill S.L. (2013). The toll and imd pathways are not required for *Wolbachia*-mediated dengue virus interference. J. Virol..

[208] Ferreira Á.G., Naylor H., Esteves S.S., Pais I.S., Martins N.E., Teixeira L. (2014). The toll-dorsal pathway is required for resistance to viral oral infection in *Drosophila*. PLoS Pathog..

[209] Chrostek E., Marialva M.S., Yamada R., O’Neill S.L., Teixeira L. (2014). High anti-viral protection without immune upregulation after interspecies *Wolbachia* transfer. PLoS ONE.

[210] Lu P., Bian G.W., Pan X.L., Xi Z.Y. (2012). *Wolbachia* induces density-dependent inhibition to dengue virus in mosquito cells. PLoS Negl. Trop. Dis..

[211] Martinez J., Tolosana I., Ok S., Smith S., Snoeck K., Day J.P., Jiggins F. (2017). Symbiont strain is the main determinant of variation in *Wolbachia*-mediated protection against viruses across *Drosophila* species. Mol. Ecol..

[212] Mosavi L.K., Cammett T.J., Desrosiers D.C., Peng Z.Y. (2004). The ankyrin repeat as molecular architecture for protein recognition. Protein Sci..

[213] Herbert R.I., McGraw E.A. (2017). The nature of the immune response in novel *Wolbachia*-host associations. Symbiosis.

[214] Bian G.W., Zhou G.L., Lu P., Xi Z.Y. (2013). Replacing a native *Wolbachia* with a novel strain results in an increase in endosymbiont load and resistance to dengue virus in a mosquito vector. PLoS Negl. Trop. Dis..

[215] McGraw E., Merritt D., Droller J., O’Neill S. (2002). *Wolbachia* density and virulence attenuation after transfer into a novel host. Proc. Natl. Acad. Sci. USA.

[216] Bull J.J., Turelli M. (2013). *Wolbachia* versus dengue: Evolutionary forecasts. Evol. Med. Public Health.

[217] Osborne S.E., Iturbe-Ormaetxe I., Brownlie J.C., O’Neill S.L., Johnson K.N. (2012). Antiviral protection and the importance of *Wolbachia* density and tissue tropism in *Drosophila simulans*. Appl. Environ.Microbiol..

[218] Weeks A.R., Turelli M., Harcombe W.R., Reynolds K.T., Hoffmann A.A. (2007). From parasite to mutualist: Rapid evolution of *Wolbachia* in natural populations of *Drosophila*. PLoS Biol..

[219] Turelli M. (1994). Evolution of incompatibility-inducing microbes and their hosts. Evolution.

[220] Hedges L.M., Brownlie J.C., O’neill S.L., Johnson K.N. (2008). *Wolbachia* and virus protection in insects. Science.

[221] Merçot H., Charlat S. (2004). Wolbachia infections in *Drosophila melanogaster* and *D. Simulans*: Polymorphism and levels of cytoplasmic incompatibility. Drosophila melanogaster, Drosophila simulans: So Similar, So Different.

[222] Micieli M.V., Glaser R.L. (2014). Somatic *Wolbachia* (Rickettsiales: Rickettsiaceae) levels in *Culex quinquefasciatus* and *Culex pipiens* (Diptera: Culicidae) and resistance to west nile virus infection. J. Med. Entomol..

[223] Sinkins S.P. (2004). *Wolbachia* and cytoplasmic incompatibility in mosquitoes. Insect Biochem. Mol. Biol..

[224] Fraser J.E., De Bruyne J.T., Iturbe-Ormaetxe I., Stepnell J., Burns R.L., Flores H.A., O’Neill S.L. (2017). Novel *Wolbachia*-transinfected *Aedes aegypti* mosquitoes possess diverse fitness and vector competence phenotypes. PLoS Pathog..

[225] Suh E., Mercer D.R., Fu Y., Dobson S.L. (2009). Pathogenicity of life-shortening *Wolbachia* in *Aedes albopictus* after transfer from *Drosophila melanogaster*. Appl. Environ. Microbiol..

[226] Kondo N., Shimada M., Fukatsu T. (2005). Infection density of *Wolbachia* endosymbiont affected by co-infection and host genotype. Biol. Lett..

[227] Dodson B.L., Hughes G.L., Paul O., Matacchiero A.C., Kramer L.D., Rasgon J.L. (2014). *Wolbachia* enhances west nile virus (WNV) infection in the mosquito *Culex tarsalis*. PLoS Negl. Trop. Dis..

[228] Joubert D.A., O’Neill S.L. (2017). Comparison of stable and transient *Wolbachia* infection models in *Aedes aegypti* to block dengue and west nile viruses. PLoS Negl. Trop. Dis..

